# Extracellular vesicles from seminal plasma interact with T cells in vitro and drive their differentiation into regulatory T‐cells

**DOI:** 10.1002/jev2.12457

**Published:** 2024-07-15

**Authors:** Xiaogang Zhang, Patrick F. Greve, Thi Tran Ngoc Minh, Richard Wubbolts, Ayşe Y. Demir, Esther A. Zaal, Celia R. Berkers, Marianne Boes, Willem Stoorvogel

**Affiliations:** ^1^ Department of Biomolecular Health Sciences Faculty of Veterinary Science Utrecht University Utrecht The Netherlands; ^2^ Department of Pediatrics and Center for Translational Immunology University Medical Center Utrecht Utrecht University Utrecht The Netherlands; ^3^ Biomolecular Mass Spectrometry and Proteomics, Bijvoet Center for Biomolecular Research and Utrecht Institute of Pharmaceutical Sciences Utrecht University Utrecht The Netherlands; ^4^ Department of Clinical Chemistry and Hematology Meander Medical Centre Amersfoort The Netherlands

**Keywords:** extracellular vesicles, immune tolerance, seminal plasma, T‐cells. Treg

## Abstract

Seminal plasma induces immune tolerance towards paternal allogenic antigens within the female reproductive tract and during foetal development. Recent evidence suggests a role for extracellular vesicles in seminal plasma (spEVs). We isolated spEVs from seminal plasma that was donated by vasectomized men, thereby excluding any contributions from the testis or epididymis. Previous analysis demonstrated that such isolated spEVs originate mainly from the prostate. Here we observed that when isolated fluorescently labelled spEVs were mixed with peripheral blood mononuclear cells, they were endocytosed predominantly by monocytes, and to a lesser extent also by T‐cells. In a mixed lymphocyte reaction, T‐cell proliferation was inhibited by spEVs. A direct effect of spEVs on T‐cells was demonstrated when isolated T cells were activated by anti‐CD3/CD28 coated beads. Again, spEVs interfered with T cell proliferation, as well as with the expression of CD25 and the release of IFN‐γ, TNF, and IL‐2. Moreover, spEVs stimulated the expression of Foxp3 and IL‐10 by CD4+CD25+CD127‐ T cells, indicating differentiation into regulatory T‐cells (Tregs). Prior treatment of spEVs with proteinase K revoked their effects on T‐cells, indicating a requirement for surface‐exposed spEV proteins. The adenosine A2A receptor‐specific antagonist CPI‐444 also reduced effects of spEVs on T‐cells, consistent with the notion that the development of Tregs and their immune suppressive functions are under the influence of adenosine‐A2A receptor signalling. We found that adenosine is highly enriched in spEVs and propose that spEVs are targeted to and endocytosed by T‐cells, after which they may release their adenosine content into the lumen of endosomes, thus allowing endosome‐localized A2A receptor signalling in spEVs targeted T‐cells. Collectively, these data support the idea that spEVs can prime T cells directly for differentiation into Tregs.

## 1 INTRODUCTION

The immune system within the female reproductive tract (FRT) is regulated by locally acting immune modulatory mechanisms. A healthy FRT, including the vagina, cervical canal, uterus, and fallopian tubes, is constitutively populated by a continuum of commensal microbiota that need to be tolerated (Chen et al., 2017). Immune tolerance towards commensal microorganisms, as well as to allogeneic sperm and to a conceptus is essential for successful fertilization and maintenance of pregnancy (Wira et al 2005); (Hansen, 2011; Nederlof et al., 2017). On the other hand, an effective adaptive immune response is required for protection against pathogenic microorganisms that may be introduced by sexual intercourse or by other means. Hereto, different tissue layers within the FRT, including submucosal tissues of the vagina, cervix, uterus, the endometrium, lamina propria and draining lymph nodes are all populated by professional antigen presenting cells (APCs), including macrophages, dendritic cells (DCs), and also major histocompatibility complex (MHC) class II expressing epithelial cells (Robertson et al., 2018). Once APCs encounter antigens within mucosal tissues, they process them internally and present fragments of these antigens on their surface using MHC molecules. At the same time, some of these activated APCs migrate to mucosal associated lymphoid tissue or secondary lymphoid organs, where they can activate cognate T‐cells to differentiate into effector T‐cells that are then instrumental for clearing the antigen bearing pathogens (Nederlof et al., 2017).

Antigens that are derived from commensal microorganisms, sperm, and the conceptus are also presented by APCs, but presentation of these antigens rather favours the differentiation of peripheral regulatory T‐cells (Tregs) that exert immune‐tolerizing functions (Robertson et al., 2018). An imbalance in effector T‐cell and Treg populations can lead to infertility, pregnancy complications, or susceptibility to infections (Huang et al., 2020). Activation and differentiation of naive T cells into Tregs is thought to involve signals from tolerogenic DCs and M2 macrophages. Epithelial cells in the cervix and uterus respond to seminal plasma by releasing cytokines, such as GM‐CSF, IL‐1β, IL‐6, and IL‐8, which facilitate the recruitment of neutrophils, macrophages, and DCs, from peripheral blood into the endometrial stroma and epithelium (De et al., 1991; Marlin et al 2019; Robertson et al., 1996). Tolerogenic DCs and M2 macrophages can then be generated in the uterus in response to soluble mediators in seminal plasma, including prostaglandin E (PGE), TLR4 ligands, transforming growth factor‐β (TGF‐β) and other cytokines (Schjenken et al., 2015). These tolerogenic APCs then migrate into draining lymphoid tissues where they may help the development of antigen specific Tregs (Robertson et al., 2018; Shima et al., 2015). Uterine tolerogenic DCs take up paternal antigens and present them to T‐cells in the draining lymph nodes, thereby inducing allogeneic paternal antigen‐specific Tregs (Yasuda et al., 2020). Paternal alloantigen specific peripheral Tregs are, however, already generated after coitus in the absence of embryo implantation (Introini et al, 2017; Robertson et al., 2013; Samstein et al., 2012; Shima et al., 2015), and expansion of Tregs and tolerance induction to paternal alloantigen can thus be driven solely by seminal fluid. Whether APCs in the mucosal tissues within the FRT can also stimulate differentiation of Tregs locally, bypassing a role for lymphoid tissues, has to our knowledge not been demonstrated. Given the successful practice of in vitro fertilization, seminal plasma may not seem to be mandatory for a successful pregnancy; however, it has been clearly demonstrated that both implantation and foetal growth are positively influenced by exposure of the mother to seminal plasma (Robertson, 2005), and prior contact with seminal fluid improves the outcome during in vitro fertilization treatment (Crawford et al., 2015). In women, seminal fluid elevates cytokine expression, immune cell recruitment and T cell activation in the cervix after coitus (Sharkey et al., 2012), and stimulates the differentiation of monocytes into tolerogenic DCs and the recruitment of Tregs into the FRT (Remes Lenicov et al., 2012; Robertson et al., 2009; Yasuda et al., 2020). Experiments with mice also demonstrated that tolerogenic DCs are recruited to the uterus and to the draining lymph nodes of the uterus, in response to coitus and independently of embryo implantation (Yasuda et al., 2020). This process is resistant to vasectomy but did not occur when the seminal glands were surgically removed (Johansson et al., 2004; Yasuda et al., 2020). These findings provide further evidence for a role of seminal plasma in promoting mating‐induced immune tolerance. Induction of male antigen‐specific tolerance by seminal plasma was further demonstrated in experiments where female mice failed to reject skin allografts of paternal origin after mating (Hancock & Faruki, 1986; Lengerova & Vojtiskova, 1963). In addition to the soluble mediators mentioned above, extracellular vesicles in seminal plasma (spEVs) also appear to play a role in immune modulation. The interaction of immune cells with spEVs protects sperm cells from being phagocytosed, damaged, or killed. For example, the activity of natural killer (NK) cells towards spermatocytes is reduced by spEVs, involving the binding of CD48 on spEVs to the NK cell activating receptor CD244 (Tarazona et al., 2011). Complement‐mediated cell lysis is also inhibited, involving CD59 and CD46 on spEVs (Kitamura et al., 1995; Rooney et al., 1993). spEVs can also interfere with mitogen‐induced lymphocyte proliferation (Kelly et al., 1991). More recently, it was demonstrated that isolated spEVs reduced the ability of antigen‐presenting cells to activate T‐cells (Vojtech et al., 2019). The term prostasomes refers to prostate‐derived EVs (Ronquist & Brody, 1985), but is often erroneously used for all spEVs. Although the majority of spEVs are derived from prostate epithelial cells (Aalberts et al. 2013a; Ronquist & Brody, 1985; Sahlén et al., 2002), other sources have also been reported, such as epithelial cells in the epididymis (Sullivan et al., 2005), seminal glands (Sahlén et al., 2010; Zhang et al., 2020a), and fractionated sperm cells (Höög & Lötvall, 2015). In the current study, we isolated spEVs from the seminal plasma of vasectomized man, to exclude any contribution from testis or epididymis, and studied their effects on the differentiation of activated T‐cells, as well as elements of the underlying molecular mechanism. We here report that naïve T cells differentiated into Tregs when activated in vitro in the presence of spEVs. Interestingly, this also occurred when isolated naïve T cells were activated with anti‐CD3/CD28 coated beads rather than with APCs, indicating a role for direct interaction of spEVs with T‐cells. Collectively, our data support the idea that spEVs may help inducing immune tolerance towards male allogens within the FRT.

## 2 MATERIALS AND METHODS

### 2.1 Isolation of spEVs from seminal plasma

Semen samples were donated as anonymized left‐over samples by vasectomized healthy men, who underwent standard laboratory confirmation following a 3‐month post‐vasectomy period, at the Andrology Laboratory of Meander Medical Centre (Amersfoort, the Netherlands). Informed consent was obtained, and the investigations were approved by the Medical Ethics Committee at Meander Medical Centre (Permission code TWO‐11‐62, 2 December 2011). Successful vasectomy (clearance) was confirmed by the absence of sperm cells in semen, as determined by phase contrast microscopy (Olympus BX40, 400×). After storage at 4°C for a maximum of 5 days, spEVs were isolated from pooled batches of seminal plasma from at least five donors. For the isolation scheme see Figure S1. Pooled seminal plasma was first diluted two‐fold with phosphate‐buffered saline (PBS, Gibco) and then centrifuged at 4°C for 10 min at 3000×g to remove any cells or cellular debris, if present. The supernatants were collected and centrifuged at 4°C for 30 min at 10,000×g to remove large microvesicles or apoptotic bodies, if present (Aalberts et al.,^2012^). The resulting supernatant was collected, and filter sterilized using a 0.2 μm filter (Millipore). The filter flow through was treated in a sterile environment from thereon, and carefully layered on top of a 1 mL 60% iohexol (Axis‐Shield, 10179579) cushion in PBS in a SW28 tube (Beckman Instruments, Inc). The tubes were topped off with PBS as required and centrifuged for 2 h at 100,000×g at 4°C in a SW28 rotor (Beckman Instruments, Inc). The iohexol cushion prevented damage or aggregation of spEVs, as may occur when EVs would be pelleted directly onto the bottom of a tube. The concentrated spEVs were visible by the naked eye as a white fluffy layer on top of the iohexol cushion. The white layer was carefully collected as a ∼1 mL sample, and mixed with 1 mL 60% iohexol to increase the sample density. The resulting ∼2 mL sample was placed at the bottom of a SW40 tube (Beckman Instruments, Inc) and overlaid with a semi‐continuous iohexol gradient. Hereto, 1 mL samples with decreasing concentrations of iohexol (45%, 40%, 35%, 30%, 25%, 20%, 15%, 10%, 5% in PBS) were carefully layered on top of each other. The gradient was finally topped off with PBS and centrifuged for 16 h at 200,000×g at 4°C in a SW40 rotor (Beckman Instruments, Inc). After centrifugation, 11 consecutive 1 mL fractions were collected from the top, ending with a 12th bottom fraction of ∼1.5 mL. Fraction densities were determined by refractometry. Iohexol is a non‐toxic, small, iodinated, high molecular weight molecule, and previously we already introduced iohexol density gradients for the isolation of EVs from blood plasma and tissue culture media (Zhang et al., 2020b). Total protein and spEVs‐associated proteins in gradient fractions were detected by 10% SDS polyacrylamide gel electrophoresis (SDS‐PAGE) and immunoblotting, as indicated below. Fractions containing spEVs were pooled, after which iohexol was removed together with any potentially remaining soluble molecules, using size exclusion chromatography (SEC). Hereto, Sepharose CL‐2B (GE healthcare, 17014001) was packed in Econo‐Pac columns (Bio‐Rad, 7321010), and equilibrated with PBS, creating 6.2 cm long columns with a diameter of 1.5 cm. After sample loading, the columns were eluted by gravity flow with PBS and 24 fractions of 500 μL each were collected. Total protein and spEVs‐associated proteins in the column fractions were detected by SDS‐PAGE and immunoblotting, as indicated below. SEC fractions containing spEVs were pooled, aliquoted, and stored at −80°C until further use. When indicated, spEVs were treated for 30 min at 37°C with 50 μg/mL proteinase K (PK, Roche, 03450376103), and then diluted with PBS containing 1 mM phenylmethylsulfonyl fluoride (PMSF, ThermoFisher, 36978) to block PK activity. Protease treated spEVs were then mixed with SDS sample buffer and immediately heated to 95°C for analysis by SDS‐PAGE and immunoblotting, or collected by ultracentrifugation for 1 h at 100,000×g at 4°C for subsequent testing on cells.

### 2.2 Analysis of spEVs by protein quantification, SDS‐PAGE, immunoblotting, transmission electron microscopy, and nanoparticle tracking analysis

Iohexol density gradients fractions and SEC columns fractions were analysed by 10% SDS‐PAGE, followed by total protein staining with Sypro ruby (Invitrogen, S12000) according to the manufacturer's instructions. Stained proteins were detected using a Bio‐Rad ChemiDoc imager. Pre‐stained protein markers (Bio‐Rad, 1610363) were used as molecular weight standards. Immunoblotting was performed as described previously (Zhang et al., 2020a). Primary antibodies include mouse anti‐human CD9 (HI9a; 312102; Biolegend; 1:2000); mouse anti‐human PSCA (clone 7F5; sc‐80654; Santa Cruz Biotechnology; 1:1000); mouse anti‐human HSP70 (N27F3‐4; ADI‐SPA‐820‐D; Enzo; 1:1000); mouse anti‐human Annexin A1 (clone 29; 610066; BD Biosciences; 1:1000); rat anti‐human Galectin‐3 (M3/38; CL8942B; CEDARLANE; 1:500) and mouse anti‐human CD47 (B6H12; sc‐12730; Santa Cruz Biotechnology; 1:500). Primary antibodies were labelled with HRP‐conjugated goat anti‐mouse IgG (Jackson immune Research; 1:10,000) or goat anti‐rat IgG (Dako; 1:5000). HRP labelled antibodies were detected using Super Signal West Dura Chemiluminescent Substrate (Thermo Fisher Scientific, 34075) and imaged and quantified using a Bio‐Rad Chemidoc system. Protein concentrations in spEVs containing fractions were determined using the Pierce BCA protein assay kit (Thermo Fisher Scientific, 23225) according to the manufacturer's protocol. Transmission electron microscopy and nanoparticle tracking analysis (NTA) of isolated spEVs were performed as described previously (Zhang et al., 2020a).

### 2.3 Labelling of spEVs with C5‐maleimide‐Alexa488

Isolated spEVs were labelled with C5‐maleimide‐Alexa488 (Invitrogen, A10254) as described (Roberts‐Dalton et al., 2017). Briefly, 100 μg samples of isolated spEVs were diluted with PBS and then concentrated by centrifugation for 2 h at 100,000×g at 4°C in a SW60 rotor (Beckman Instruments, Inc). Pelleted spEVs were resuspended in 50 μL PBS, mixed with 5 μL C5‐maleimide‐Alexa488 (200 μg/mL), and incubated for 2 h at room temperature while being shielded from light. After incubation, samples were diluted with 4 mL excess volume PBS containing 0.1% bovine serum albumin (BSA), and centrifuged for 2 h at 100,000×g 4°C in a SW60 rotor. For this purpose, BSA was prepared as a 100 mg/mL stock solution that had been centrifuged for 2 h at 100,000×g 4°C in a SW60 rotor to remove any insoluble material, and was filter sterilized. To remove any remaining free dye, pelleted Alexa488‐labelled spEVs (Alexa488‐spEVs) were washed with PBS/0.1% BSA using the same centrifugation procedure, and finally resuspended in 100 μL of the same buffer. Alexa488‐spEVs were aliquoted and stored at −80°C until use. Negative control samples were generated by mixing the same amount of C5‐maleimide‐Alexa488 with PBS in the absence of spEVs, followed by identical processing as the Alexa488‐spEVs containing samples.

### 2.4 Cell culture and flow cytometric analysis

Blood was obtained from healthy volunteers following institutional ethical approval (www.umcutrecht.nl/METC; protocol number 07–125/C). Peripheral blood mononuclear cells (PBMCs) were isolated from lithium‐heparinized whole blood samples using Ficoll isopaque density gradient centrifugation (GE Healthcare). Cells were cultured in 96‐well plates at 37°C and 5% CO_2_ in RPMI 1640 GlutaMAX (Gibco, 61870036) supplemented with 1% Penicillin/Streptomycin (Gibco) and 10% EV‐free foetal calf serum (FCS). EV‐free FCS was prepared by diluting FCS to 30% with RPMI 1640 GlutaMAX, followed by ultracentrifugation in a SW27 rotor for 16 h at 100,000×g at 4°C, discarding the pellet together with the bottom 5 mL of the tube, and 0.2 μm filtration.

For mixed lymphocyte reaction (MLR) experiments, CD3+ cells were separated from the PBMCs using CD3+ MicroBeads (Miltenyi Biotec, 130‐050‐101) and manual MACS with MS separation columns (Milteny Biotec, 130‐042‐201) according to the manufacturer's protocol. The CD3+ cell fraction was subsequently stained with 2 μM Cell Trace Violet (CTV) (Fisher Scientific, 15579992) for 30 min at room temperature and then washed with FCS. PBMCs from HLA‐mismatched donors were obtained from frozen PBMC stocks. The freshly isolated CD3+ lymphocytes and thawed PBMCs were resuspended to a concentration of 1 × 10^6^ cells/mL each in RPMI 1640 GlutaMAX (Gibco) supplemented with 1% penicillin/streptomycin, 1% L‐glutamine and 10% foetal calf serum. For direct concurrent incubation with spEVs, 1 × 10^5^ CD3+ cells were plated in 96‐well plates followed by adding 1 × 10^5^ thawed PBMCs. As a negative control, 100 μL of medium lacking PBMCs was added instead. Immediately thereafter, isolated spEVs in PBS were added to a final concentration of 60 μg/mL. As a negative control, an equal amount of PBS was added instead. Cells were cultured for 4 days at 37°C before flow cytometry analysis. When indicated, spEVs preincubation conditions were used. Hereto CD3+ cells and PBMCs were plated separately in a 96‐wells plate, and spEVs were added at 60 μg/mL to the CD3+ cells only. CD3+ cells were incubated for 16 h with spEVs, and then washed with excess culture medium by centrifugation to remove non cell‐associated spEVs, and subsequently cultured together with the PBMCs for four days. After culture, cells were stained with eFluor 780 viability dye (eBioscience, 65‐0865‐14) for 20 min at 4°C. Cells were washed with flow cytometry buffer and subsequently labelled with FITC‐conjugated mouse anti‐human CD3 (clone OKT3; 1:25; Biolegend), PE‐conjugated mouse anti‐human CD4 (clone RPA‐T4; 1:100; Biolegend) and PE‐Cy7‐conjugated mouse anti‐human CD8 (clone SK1; 1:200; BD). After 15 min at 4°C, the cells were washed again and resuspended in flow cytometry buffer for analysis using a BD LSRFortessa flow cytometer, and the data were analysed with FlowJo software (Treestar, version 10.0).

For anti‐CD3/CD28 driven T‐cell activation experiments, pan T‐cells were isolated from freshly isolated PBMCs by negative selection using a pan T‐cell isolation kit (Miltenyi Biotec, 130096535), according to manufacturer instructions. The purity of the isolated pan T‐cells was evaluated using flow cytometry (see below), and only isolates containing >95% CD3+ cells were used for experiments. Pan T‐cells were cultured in 96‐well plates at a concentration of 5 × 10^5^ cells/mL in RPMI 1640 GlutaMAX supplemented with 10% heat‐inactivated EV‐free FCS and 1% Penicillin/Streptomycin. For pan T‐cell proliferation experiments, isolated pan T‐cells were first labelled with Cell Trace Violet (CTV) (Thermo Fisher Scientific, C34557). Hereto, pan T‐cells were pelleted and resuspended in PBS containing 2.5 μM CTV, and incubated for 20 min at 37°C. Then, 10 mL excess complete culture media was added, and CTV‐labelled pan T‐cells were pelleted and resuspended in complete culture media. Pan T‐cells were stimulated using Dynabeads coated with both CD3 and CD28‐directed antibodies (Gibco, 11131D), either in the absence or presence of spEVs, as indicated. Conditioned cell culture medium was collected, and cytokines were detected by ELISA as described below. Cells were collected by centrifugation and analysed by flow cytometry. For pan T‐cell proliferation assays, CTV labelled cells were stained with FITC‐conjugated mouse anti‐human CD3 (OCT‐03; 1:50; Biolegend), APC‐eFluor780‐conjugated mouse anti‐human CD4 (RPA‐T4; 1:50; eBioscience), and APC‐conjugated mouse anti‐human CD8 (RPA‐T8; 1:50; eBioscience). To detect apoptotic and dead T‐cells, pan T‐cells were resuspended in binding buffer supplemented with APC‐labelled Annexin V (1:20; BD) and 7‐AAD (1:20; BD), incubated for 15 min at room temperature, and supplemented with excess binding buffer before analysis by flow cytometry. To detect Treg, T‐cells were first stained with PB‐conjugated mouse anti‐human CD3 (UCHT1; 1:100; Sony Biotechnology), PE‐conjugated mouse anti‐human CD4 (RPA‐T4; 1:50; BD), FITC‐conjugated mouse anti‐human CD25 (M‐A251; 1:50; BD), and PE‐Cy‐conjugated mouse anti‐human CD127 (HCD127; 1:50; eBioscience). Subsequently, these cells were washed with PBS, fixed for 10 min with 4% paraformaldehyde in PBS, washed with PBS, permeabilized with PBS containing 0.1% saponin and 0.5% BSA, blocked for 15 min in PBS containing 0.1% saponin, 0.5% BSA and 5% normal mouse serum or PBS containing 0.1% saponin, 0.5% BSA and 5% normal rat serum, and finally labelled with AF647‐conjugated mouse anti‐human Foxp3 (MOPC‐21; 1:25; Biolegend), PE‐conjugated rat anti‐human IL‐10 (JES3‐19F1; 1:25; BD), or PB‐conjugated rat anti human IL‐2 (MQ1‐17H12; 1:100; Biolegend) in blocking buffer. Labelled cells were then washed twice with permeabilization buffer, and resuspended in flow cytometry buffer. Labelling specificity of all antibodies was controlled using isotype control‐matched antibodies. Flow cytometry was performed on a FacsCanto II (BD) flow cytometer and the data were analysed with FlowJo software (Treestar, version 10.0).

To measure the recruitment of spEVs by flow cytometry, PBMCs or pan‐isolated T‐cells were incubated in the presence of 30 μg/mL Alexa488‐spEVs, or an equal volume of dye control (see above). After incubation, PBMCs were washed once with culture media, and twice with flow cytometry buffer (PBS with 0.5% BSA and 0.02% NaN_3_). Then, the cells were blocked in flow cytometry buffer supplemented with 5% normal mouse serum (Fitzgerald, 88R‐M002) and labelled with Pacific Blue (PB)‐conjugated mouse anti‐human CD3 (clone UCHT1; 1:50; Sony Biotechnology), PB‐conjugated mouse anti‐human CD14 (clone HCD14; 1:50; Sony Biotechnology), PB‐conjugated mouse anti‐human CD19 (clone HIB19; 1:50; Biolegend), or Allophycocyanin (APC)‐conjugated mouse anti‐human CD56 (clone B159; 1:50; BD). 7‐AAD (1:50; BD) was used to label dead cells. Pan T‐cells loaded with Alexa488‐spEVs were labelled with FITC‐conjugated mouse anti‐human CD3 (OKT3; 1:50; Biolegend), PerCP‐conjugated mouse anti‐human CD4 (RPA‐T4; 1:25; Biolegend), V500‐conjugated mouse anti‐human CD8 (RPA‐T8; 1:25; BD), PE‐conjugated mouse anti‐human CD25 (M‐A251; 1:20; BD), and APC‐conjugated mouse anti human‐CD69 (FN50; 1:20; BD). Flow cytometry was performed on a FacsCanto II (BD) flow cytometer and the data were analysed with FlowJo software (Treestar, version 10.0).

### 2.5 Live cell confocal fluorescence microscopy

PBMCs or pan T‐cells were incubated for 16 h with 30 μg/mL Alexa488‐spEVs, or an equal volume of dye control (see above). Cells were then harvested and washed with culture media to remove free spEVs, and plated in a CELLview 4 well glass bottom cell culture dish (Greiner) at 5 × 10^5^ cells/mL. When indicated, the cell medium was supplemented with lysotracker RED DND‐99 (1:1000; Thermo Fisher Scientific, L7528). Still images of confocal sections of live cells were made with a NIKON AIR confocal microscope using a 60× Plan Apo oil immersion objective (N.A. 1.4) with laser and filter settings for Alexa488 and TxR fluorescence detection while maintaining cell culture conditions (37°C and 5% CO_2_) in a tabletop culture control unit (TOKAI Hit) equipped with a lens heater module. For imaging of live whole cells, confocal sections with a depth of 0.3 μm were stacked and analysed using NIS‐elements imaging software (Nikon, version 5.20).

### 2.6 Cytokine detection by ELISA

Human IFN‐γ, TNF, and IL‐2 in collected cell conditioned culture media were determined using ELISA kits for human IFN‐γ (Thermo Fisher Scientific, 88731688), TNF (Thermo Fisher Scientific, 88734688), and IL‐2 (Biolegend, 431815) according to manufacturers’ protocols.

### 2.7 LC‐MS

Two hundred microgram samples of isolated spEVs were diluted to 1 mL with PBS, pelleted by ultracentrifugation at 100,000×g for 2 h at 4°C using a TLA55 rotor and Beckman–Coulter Optima MAX‐XP ultracentrifuge, and lysed in 50 μL ice cold lysis buffer (2:2:1 methanol/acetonitrile/ultrapure LC‐MS‐grade water), shaken for 10 min at 4°C, and centrifuged at 14,000×g for 15 min at 4°C. Five microlitre samples of supernatant were subjected to LC‐MS. LC‐MS analysis was performed on an Exactive mass spectrometer (Thermo Scientific) coupled to a Dionex Ultimate 3000 autosampler and pump (Thermo Scientific). The MS was operated in polarity‐switching mode with spray voltages of 4.5 and −3.5 kV. Metabolites were separated using a Sequant ZIC‐pHILIC column (2.1 × 150 mm, 5 μm, guard column 2.1 × 20 mm, 5 μm; Merck) using a linear gradient of acetonitrile and eluent A (20 mM (NH4)2CO3, 0.1% NH4OH in ultrapure LC‐MS‐grade water; Biosolve). The flow rate was set at 150 μL/min. Metabolites were identified on positive mode based on exact mass within 5 ppm and were validated by comparison with retention times of standards. Metabolites were quantified and analysed with TraceFinder 5.0 software (Thermo Scientific). Represented data were based on the peak area of identified metabolites.

### 2.8 Statistical analysis

Statistical analysis was performed using GraphPad Prism 8.0 software. Paired t tests were used to compare two groups, and paired one‐way ANOVA for >2 groups. *p* ≤ 0.05 was considered statistically significant. *, **, *** and **** indicate *p* < 0.05, *p *< 0.01, *p* < 0.001 and *p* < 0.0001, respectively.

## 3 RESULTS

### 3.1 spEVs isolation and characterization

**FIGURE 1:**
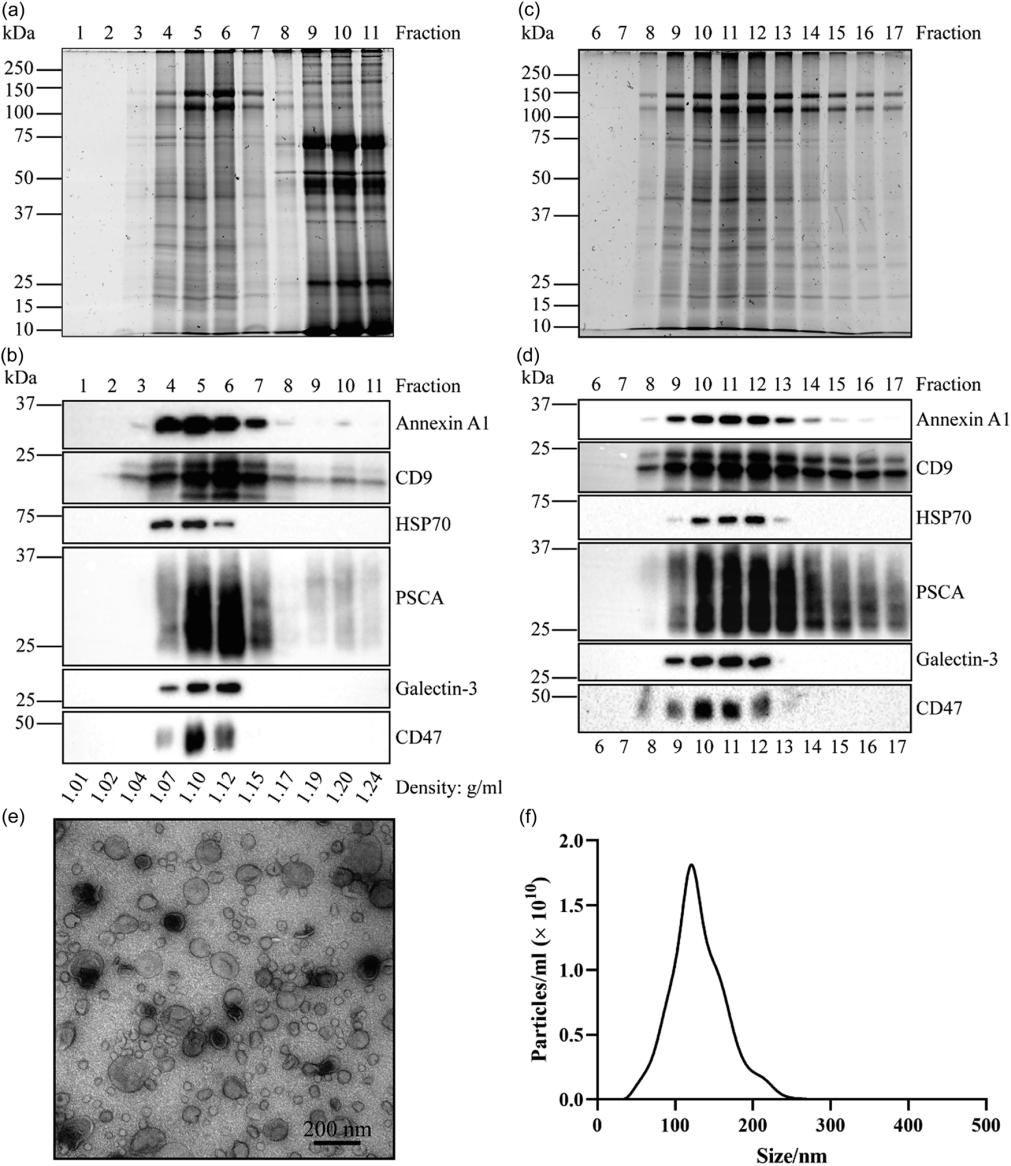
Isolation and characterization of spEVs. spEVs were collected from pooled seminal plasma samples from vasectomized men by ultracentrifugation on top of an iohexol cushion, and then loaded at the bottom of an iohexol density gradient and floated upward into the gradient by ultracentrifugation. Gradient fractions were collected from the top and analysed by SDS‐PAGE, followed by total protein staining (a), or immunoblotting for the presence of Annexin A1, CD9, HSP70, PSCA, Galectin‐3, and CD47 (b). Molecular weight markers are indicated on the left in kDa. Density gradient fractions containing spEVs (4‐7) were pooled and spEVs isolated further by SEC. SEC fractions 6‐17 were analysed by SDS‐PAGE followed by total protein staining (c) or immunoblotting for the presence of Annexin A1, CD9, HSP70, PSCA, Galectin‐3 and CD47 (d). Molecular weight markers are indicated on the left in kDa. SEC fractions containing isolated spEVs (9‐13) were pooled and analysed by transmission electron microscopy (e) and NTA (f).

spEVs were isolated from the seminal plasma of vasectomized men to exclude contributions of EVs from the testis and epididymis. Previously, we already demonstrated that spEVs isolated from seminal plasma of vasectomized men originated in majority from the prostate, although contributions from seminal glands were also detected (Aalberts et al., 2013a; Zhang et al., 2020a). In these studies, we separated two differently sized populations of spEVs, with average diameters of 50 and 100 nm, by making use of their distinct migration velocities during sucrose density gradient centrifugation. In some studies, it has, however, been observed that the separation of EVs on sucrose density gradients may interfere with their biological activities (Reiner et al., 2017). To allow analysis of the effects of spEVs on immune cells, we therefore chose to modify our isolation procedure (See Materials and Methods section and Figure S1), by replacing sucrose density gradients with iohexol density gradients. After ultracentrifugation, gradient fractions were collected and analysed by SDS‐PAGE followed by total protein staining and immunoblotting. spEVs floated to fractions 4–7 at their equilibrium density between 1.07 and 1.15 g/mL (Figure 1a, b). Proteins that were not associated with spEVs were retained in bottom fractions 9–11 (Figure 1a). EV markers, including Annexin A1, CD9, HSP70, Galectin‐3, CD47, and the prostate‐specific EV marker PSCA were detected in fraction 4–7 (Figure 1b). To remove iohexol and any potentially remaining soluble contaminants, pooled density fractions 4–7 were loaded onto an SEC column, and eluted with PBS. All EV markers and most non‐specified proteins eluted in SEC fractions 9–13 (Figure 1c, d). Purified spEVs from SEC fractions 9–13 were pooled and analysed further by transmission electron microscopy and NTA. According to transmission electron microscopic images, the majority of spEVs varied in diameter from 50 to 150 nm (Figure 1e), consistent with our previous reports (Aalberts et al., 2012; Zhang et al., 2020a). The NTA data indicate a mean size of 131 nm for detectable spEVs (Figure 1f), but it should be noted that detection by NTA of small (< 100 nm) is less efficient compared to larger (> 100 nm) EVs. The entire isolation procedure delivered highly purified spEVs, with a ∼ 60% yield (Figure S1C). Taking into account a 40% loss, we calculated that seminal plasma of vasectomized men contained 0.4 ± 0.1 mg/mL spEVs associated protein, representing 6 × 10^12^ ± 1.2 × 10^12^ NTA detectable spEVs/mL. However, given that EVs smaller than 100 nm are inefficiently detected by NTA, the true number of spEVs in seminal plasma is underestimated by this method.

### 3.2 spEVs inhibit T‐cell proliferation in MLR

**FIGURE 2:**
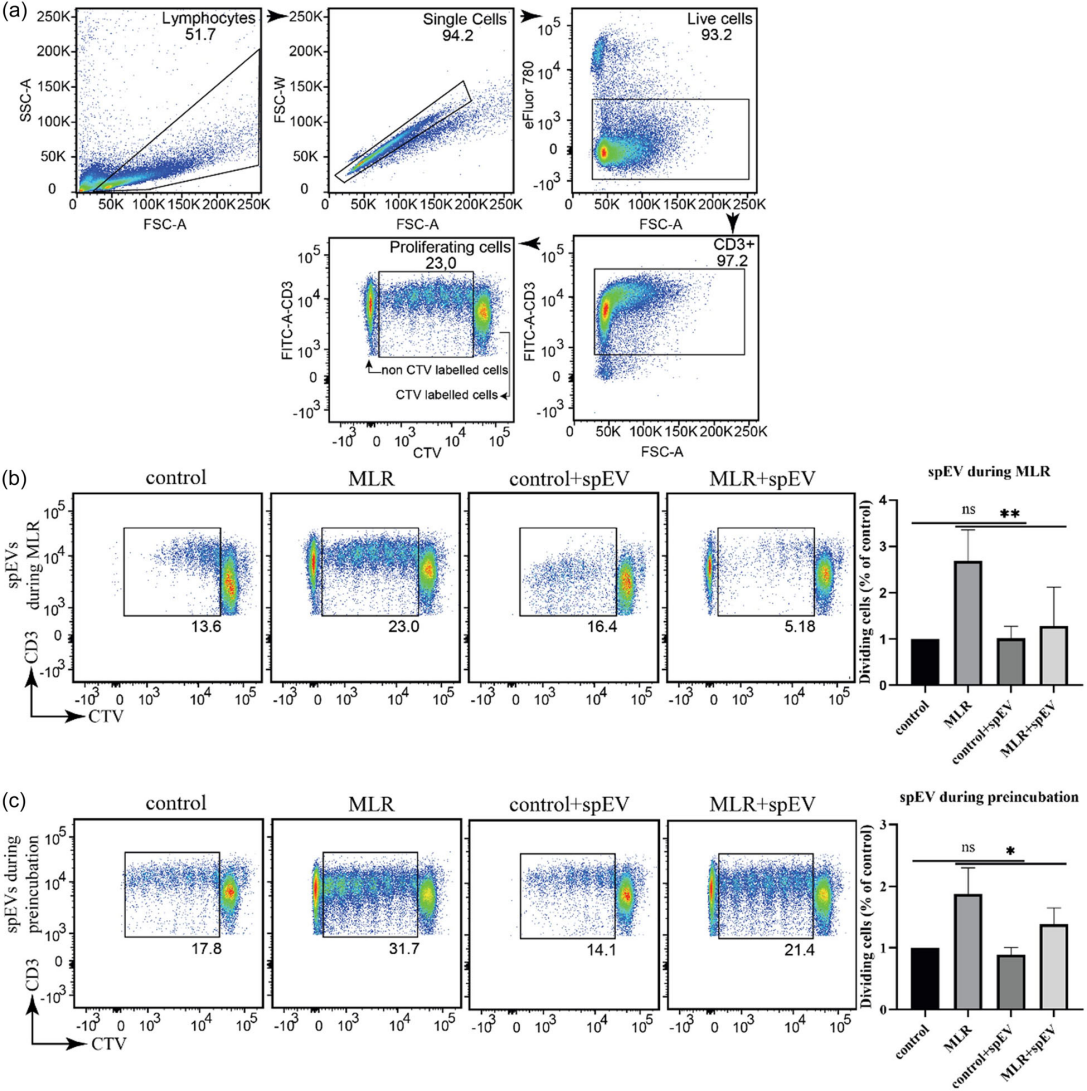
spEVs inhibit T‐cell proliferation in MLR. Proliferation of isolated and CTV labelled CD3+ T‐cells in response to HLA‐mismatched PBMCs was detected by dilution of the CTV dye. (a), Representative dot plots representing the gating strategy for the detection of live single CTV labelled proliferating CD3+ T cells after MLR. (b, c), The presence of spEVs interfered with the proliferation of CTV labelled CD3+ T‐cells during MLR. CTV‐labelled CD3+ T cells were cultured either in the presence or absence of mismatched unlabelled PBMCs, and in the presence or absence of spEVs, as indicated. spEVs were either added concomitantly with the mismatched PBMCs (b), or present only during a preincubation period of 16 h prior to the addition of mismatched PBMCs (c). The CTV negative cells in the MLR samples represent T‐cells from the mismatched unlabelled PBMCs. The dot plots are representative examples of four independent experiments. The histograms in the right panels in B and C indicate the fraction of dividing CTV labelled T‐cells at each condition from four independent experiments (mean ± SD, *n* = 4).

Potential immunomodulatory effects of spEVs were first tested using MLR, a readout for T‐cell activation that occurs when T‐cells are co‐cultured with antigen‐presenting cells from an allogeneic donor. As a read‐out for this activation, we measured the proliferation of isolated CTV‐labelled CD3+ T‐cells that were co‐cultured for 4 days with HLA‐mismatched PBMCs (Figure 2). T‐cell proliferation was detected by flow cytometry, measuring the dilution of the CTV dye. As expected, in the absence of PBMCs (negative control), only 15.3 ± 4.0% of the CD3+ T‐cells proliferated (*n* = 4 independent donors; Figure 2b). In the presence of allogeneic PBMCs, the amount of proliferating CD3+ T‐cells increased to 36.1 ± 12.7%, depending on the HLA‐mismatch with the donor. Stimulation of T‐cell proliferation by allogeneic PBMCs was significantly reduced in the concomitant presence of spEVs (Figure 2b). The same inhibitory effect was observed when T‐cells were first incubated for 16 h with spEVs, then washed to remove excess spEVs, and subsequently incubated for 4 days with allogeneic PBMCs (Figure 2c). These data indicate that spEVs intervened with PBMC‐driven T‐cell activation by preconditioning of the T‐cells rather than by interfering with the function of the T‐cell activating allogeneic APCs present in PBMCs.

### 3.3 Recruitment and endocytosis of spEVs by PBMCs

**FIGURE 3:**
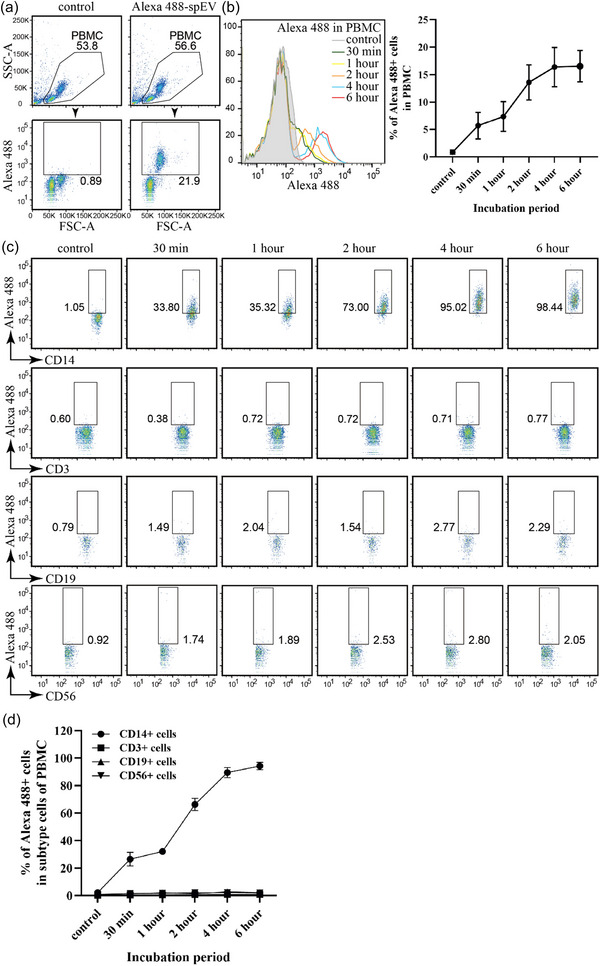
In PBMCs, spEVs are recruited mainly by CD14+ macrophages. Isolated PBMCs were cultured in the absence (control) or presence of Alexa 488‐spEVs, as indicated, and thereafter analysed by flow cytometry. (a) Representative dot plot diagrams demonstrating the gating strategy used to determine cells in PBMCs that recruited Alexa 488‐spEVs after 6 h incubation. (b) Representative flow cytometry histograms of PBMCs, demonstrating increasing uptake of Alexa 488‐spEVs during 0–6 h incubation (left panel) and the quantification of Alexa 488‐spEVs labelled PBMCs (right panel; mean ± SD, from four independent experiments). (c) Representative experiment showing dot plot diagrams of Alexa 488‐spEV positive CD14+, CD3+, CD19+ or CD56+ gated cells, after 6 h incubation. (d) Quantification of data as in C (mean ± SD, *n* = 4).

**FIGURE 4:**
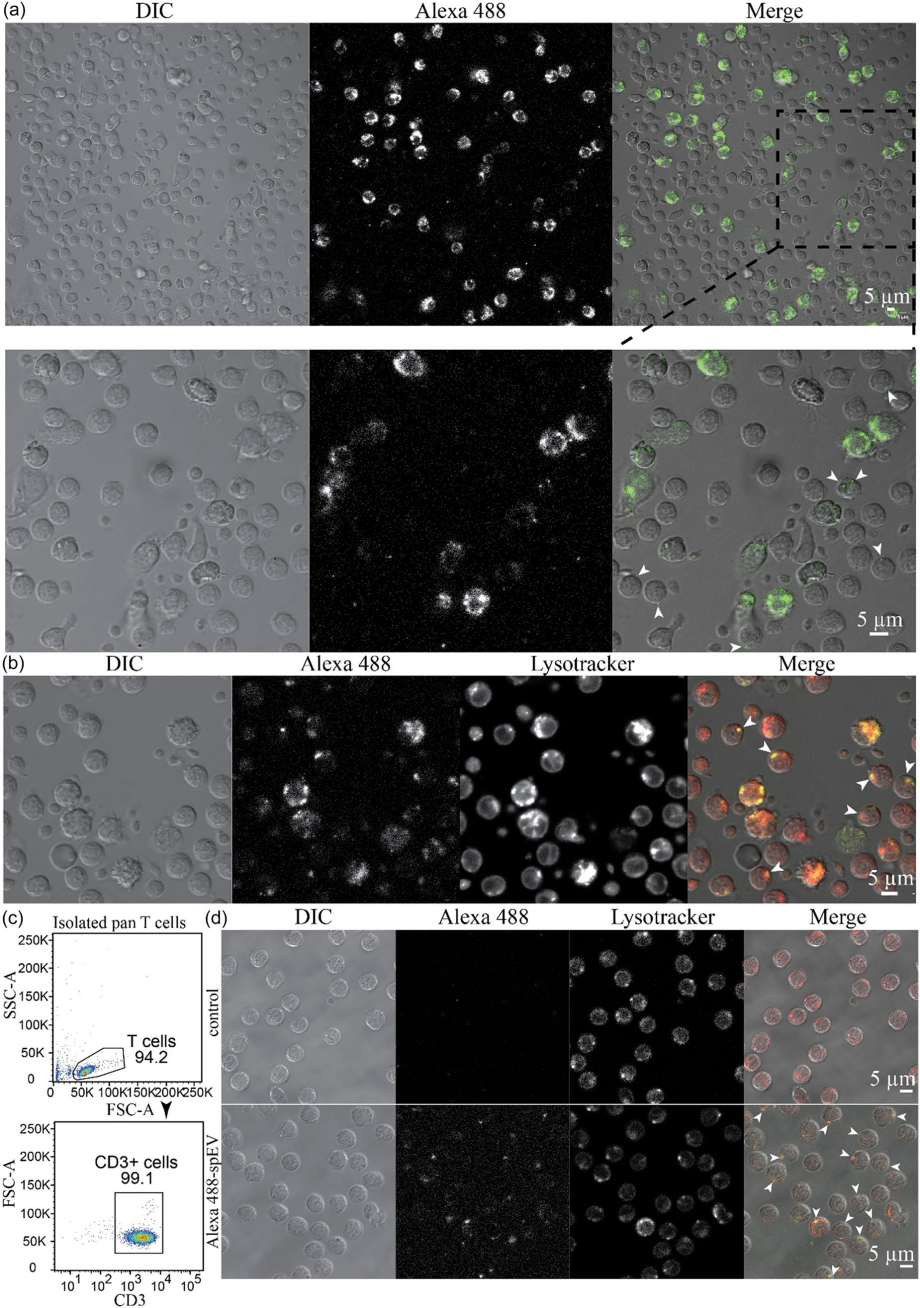
spEVs are endocytosed by both macrophages and T‐cells. (a) PBMCs were cultured for 16 h in the presence of Alexa 488‐spEVs, washed, and imaged directly by live cell confocal fluorescence microscopy. Differential interference contrast (DIC), Alexa488 spEVs, and merged channels are displayed as indicated. The second row are enlargements of the indicated area in the top row. Note the intensely labelled large cells (macrophages), and much less but distinct labelling at discrete puncta within smaller cells. **(b)** Alexa 488‐spEV labelled cells were imaged in pre‐warmed culture media containing lysotracker and imaged by live cell confocal fluorescence microscopy. Note that the Alexa488‐spEV positive spots coincide with lysotracker positive endocytic compartments. Arrowheads in the right panel indicate smaller‐sized cells in PBMCs that contain only a few and small Alexa 488‐spEV positive compartments, also colocalizing with lysotracker. These data are representative of three independent experiments. Bars, 5 μm. **(c)** Pan CD3+ T‐cells were isolated from PBMCs, and their purity is represented by a flow cytometry dot plot. **(d)** Isolated pan T‐cells were cultured for 16 h in the absence (control) or presence of Alexa488‐spEVs, washed with culture media to remove non‐bound Alexa 488‐spEVs, supplemented with lysotracker and imaged directly at T‐cell culture conditions by live cell confocal fluorescence microscopy. DIC, Alexa 488‐spEVs, lysotracker, and merged images are as indicated. Alexa 488‐spEVs are contained by a limited number of small lysotracker‐positive endocytic compartments (indicated by arrowheads). These data are representative of three independent experiments. Bars, 5 μm.

To demonstrate the recruitment of spEVs by cells, isolated spEVs were first fluorescently labelled by covalent linkage of Alexa488 C5‐maleimide to thiol groups of surface‐exposed proteins. This procedure has several advantages over labelling EVs with lipophilic dyes such as PKH. In contrast to lipophilic dyes, non‐bound Alexa488 C5‐maleimide does not form micelle‐like aggregates in aqueous solutions, and thus can be more easily removed from labelled EVs. Moreover, covalently bound dyes cannot be exchanged between EVs and cell membranes, while lipophilic dyes such as PKH can be exchanged, without actual EV transfer, resulting in false positive signals for EV uptake by cells (Simonsen, 2019). PBMCs were cultured for the indicated time in the presence of Alexa488‐spEVs and then analysed by flow cytometry for associated Alexa488‐spEVs (Figure 3). Alexa488‐spEVs were detected on 16.3 ± 3.6% of PBMCs, reaching a plateau at 4 h (Figure 3a, b). PBMCs failed to recruit Alexa488‐spEVs at 4°C (Figure S2), indicating temperature‐dependence for spEV binding or endocytosis. Next, we identified those cell types in PBMCs that acquired Alexa488‐spEVs with high efficiency (Figure 3c–e). With this approach, we found that Alexa488‐spEVs were recruited predominantly by CD14+ monocytes. In comparison, CD3+ T‐cells, CD19+ B cells, and CD56+ NK cells acquired much less if any Alexa488‐spEVs, as detected by flow cytometry. To confirm these data and determine whether recruited spEVs were endocytosed, we used confocal fluorescence microscopy (Figure 4). As measured by this approach also, Alexa488‐spEVs were predominantly recruited by a selective population of relatively large PBMCs (Figure 4a). Lysotracker was used to label acidic organelles in live cells, including late endosomes and lysosomes (Majzoub et al., 2016). Interestingly, the majority of the acquired Alexa488‐spEVs colocalized with lysotracker, indicating endocytic uptake (Figure 4b). Moreover, by using confocal fluorescence microscopy, we found an additional population of relatively smaller cells in PBMCs that contained endocytosed Alexa488‐spEVs, albeit less in comparison to the larger monocytes (Figure 4b, indicated by arrowheads), and again coinciding with lysotracker. This signal was apparently too low for (non‐spatial) detection by flow cytometry. To determine whether T‐cells could endocytose Alexa488‐spEVs, CD3+ T‐cells were isolated from PBMCs by autoMACS and then incubated for 16 h with Alexa488‐spEVs. Analysis of the isolated T cells by flow cytometry indicate the purity of the T cell isolate (Figure 4c). In confocal sections, most T‐cells (55%, *n* = 200) displayed either one or two small intracellular loci that were positive for both Alexa488 and lysotracker, indicative for spEVs containing endosomes or lysosomes (Figure 4d). In 3D profiles (Z‐stacked confocal sections), all analysed T‐cells contained compartments labelled with both Alexa488 and lysotracker (Video S1).

### 3.4 spEVs inhibit T‐cell function and promote Treg differentiation

**FIGURE 5:**
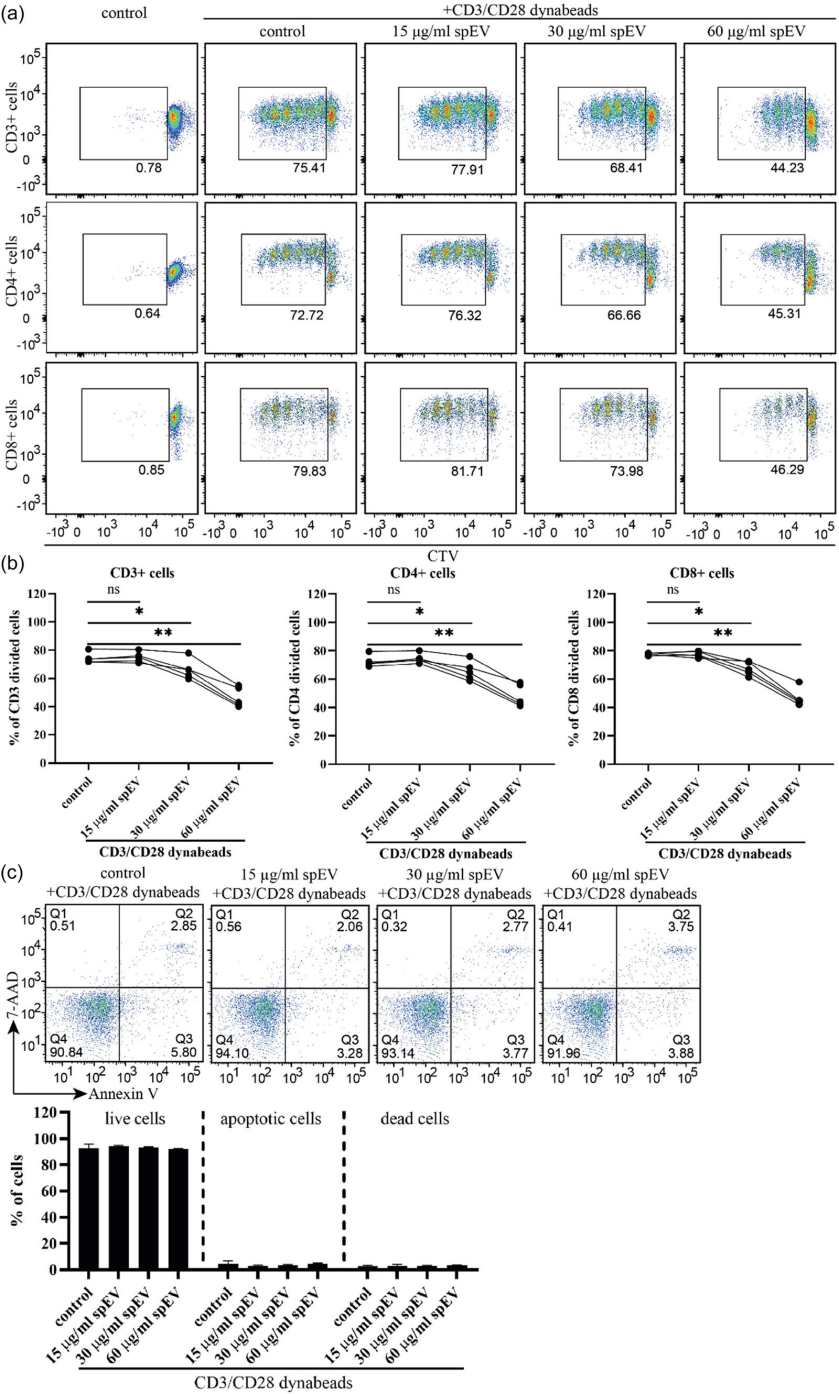
spEVs interfere with proliferation of isolated anti‐CD3/CD28 activated T‐cells. Pan T‐cells were isolated from PBMCs, labelled with CTV, and stimulated for 4 days with anti‐CD3/CD28 Dynabeads in the absence (control) or presence of different concentrations of spEVs, as indicated. The absence of anti‐CD3/CD28 Dynabeads was used as a negative control. (a) Representative flow cytometry dot plots demonstrating proliferation of pan CD3+ T‐cells as well as CD4+ and CD8+ T‐cells, by measuring CTV dilution. (b) Quantification of proliferated CD3+, CD4+, or CD8+ cells from 4 independent experiments as shown in A. (c) Isolated pan T‐cells were stimulated with anti‐CD3/CD28 Dynabeads in the absence (control) or presence of different concentrations of spEVs, as indicated, labelled with Annexin V and 7‐AAD, and analysed by flow cytometry for the presence of apoptotic cells (Annexin V+) and dead cells (7‐AAD+). The dot plots show a representative experiment, histograms show the cumulation of quantitative data from four independent experiments (mean ± SD).

After having identified an interaction of spEVs with T‐cells, we continued investigating direct effects of spEVs on T‐cell differentiation in the absence of APCs. (Figure 5). Hereto, isolated naïve pan T‐cells were first labelled with CTV, and then stimulated with anti‐CD3/CD28 coated Dynabeads in the absence or presence of spEVs. After 4 days of culturing, T‐cell proliferation was detected by flow cytometry, measuring CTV dilution. (Figure 5a, b; for gating strategy of CD3+ total, CD4+, and CD8+ T cells see Figure S3). spEVs inhibited CD4+ T‐cell proliferation down to 48 ± 8%, and CD8+ T‐cell proliferation down to 47 ± 6%, in a dose‐dependent manner. To control for cell death and apoptosis, treated T‐cells were labelled with Annexin V and 7‐AAD, and analysed by flow cytometry (Figure 5c). spEVs did not induce T‐cell death or apoptosis.

**FIGURE 6:**
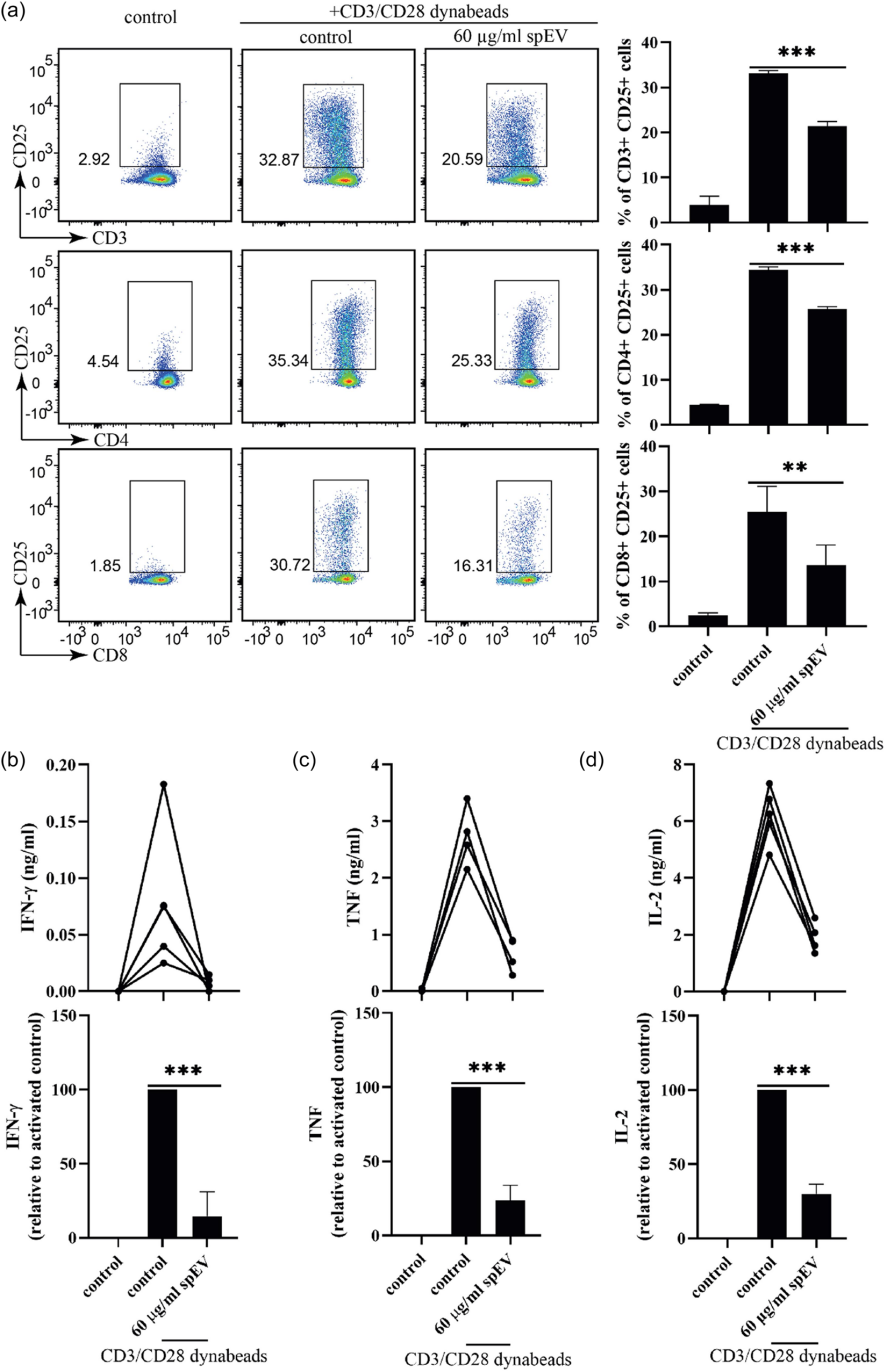
spEVs interfere with the induction of T‐cell activation markers and cytokine production. (a) Isolated pan T‐cells were either left untreated (control) or stimulated for 16 h by anti‐CD3/CD28 Dynabeads in the absence (control) or presence of 60 μg/mL spEVs. Expression of the T‐cell activation marker CD25 on CD3+ pan T‐cells, CD4+ T‐cells and CD8+ T‐cells was then analysed by flow cytometry. A representative example is presented by the dot plots in the left panels. Boxed areas indicate CD25‐high T‐cells. The histograms in the right panels show the quantification of four independent experiments (mean ± SD, *n* = 4). (b–d) Cell culture media from the experiments in A were collected and analysed for the presence of IFN‐γ, TNF and IL‐2 by ELISA. The concentrations of IFN‐γ, TNF and IL‐2 are plotted both in ng/mL and percentage relative to control (mean ± SD, *n* = 4 independent experiments).

As determined by surface expression of the T‐cell activation marker CD25, ∼35% of both CD4+ and CD8+ T‐cells were activated after 16 h incubation with anti‐CD3/CD28 coated Dynabeads in the absence of spEVs (Figure 6a; for gating strategy see Figure S3). The concomitant presence of spEVs significantly reduced the percentages of CD25+ cells in such activated CD4+ and CD8+ T‐cell populations. Moreover, the bead induced release of the cytokines IFN‐γ, TNF, and IL‐2, by anti‐CD3/CD28 stimulated T‐cells was strongly inhibited by the presence of spEVs, down to 14 ± 17%, 24 ± 10%, and 30 ± 7%, respectively (Figure 6b–d). The inhibitory effects on cytokine production and CD25 expression were spEVs concentration‐dependent (Figures S4 and S5). IL‐2 production was equally reduced in CD3+, CD4+, and CD8+ cells, as determined by flow cytometry (Figure S4A). Interference by spEVs of cytokine release and CD25 expression was sustained after 72 h of T‐cell stimulation (Figure S6). Collectively, these data demonstrate that spEVs have potent and sustained inhibitory effects on T‐cell activation, as determined by T‐cell proliferation, expression of the activation marker CD25, and cytokine production.

**FIGURE 7:**
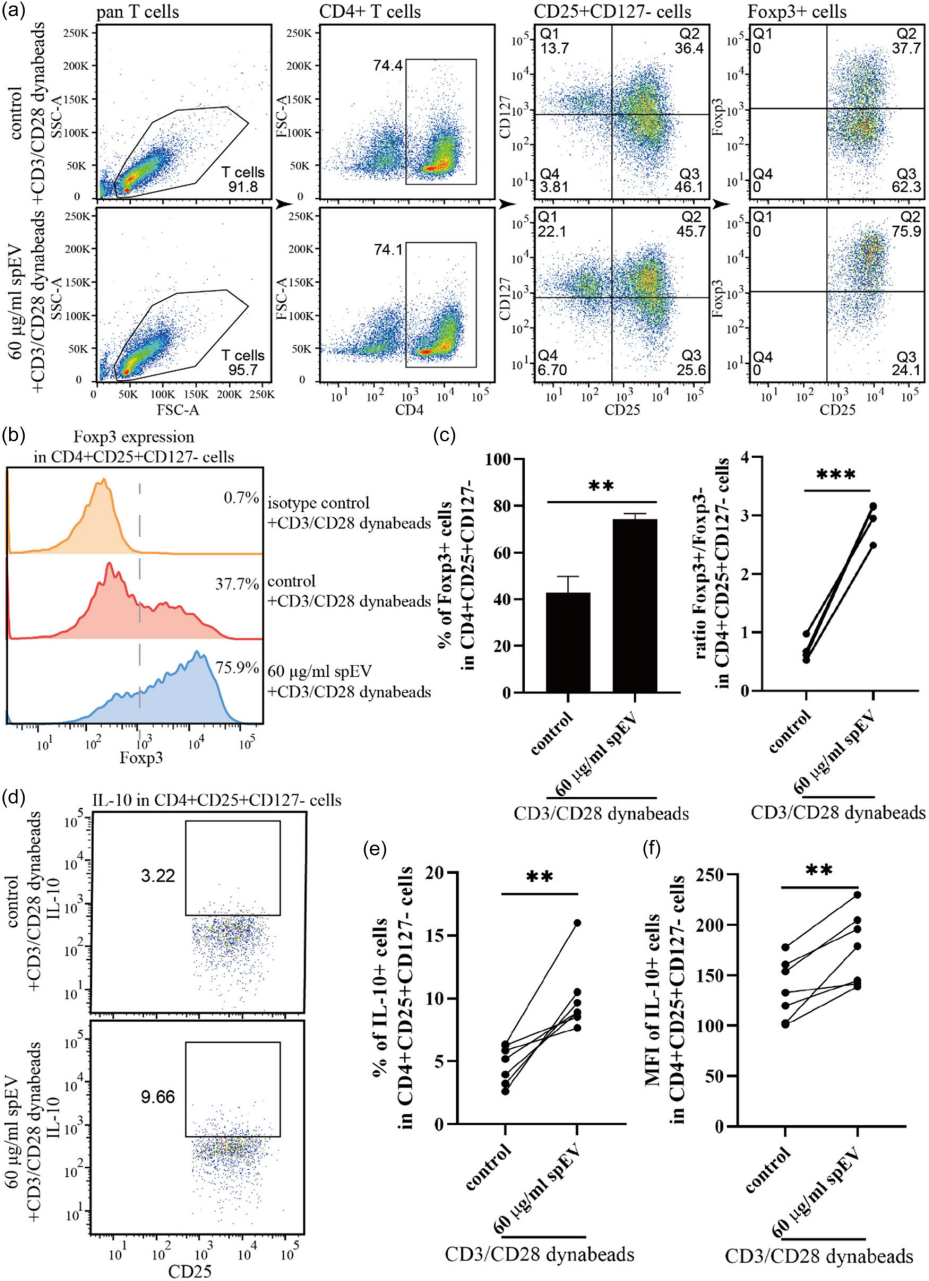
spEVs promote the development of functional Tregs. Isolated pan T‐cells were stimulated for 4 days by anti‐CD3/CD28 Dynabeads, either in the absence (control) or presence of 60 μg/mL spEVs, after which expression of Foxp3 in CD3+CD4+CD25+CD127‐ cells was determined by flow cytometry. (a) Representative dot plots demonstrating the gating strategy. (b) Representative histograms indicating Foxp3 expression in CD4+CD25+CD127‐ gated cells. (c) Quantification of data as in B as a percentage of Foxp3+ cells (left panel, mean ± SD) and the ratio of Foxp3+/Foxp3‐ cells in population of CD3+CD4+CD25+CD127‐ gated cells (right panel), for four independent experiments. (d–f) To demonstrate that spEVs promoted the generation of functional Tregs, isolated pan T‐cells were stimulated for 4 days by anti‐CD3/CD28 Dynabeads, either in the absence (control) or presence of 60 μg/mL spEVs, after which IL‐10 expression in CD3+CD4+CD25+CD127‐ cells was analysed by flow cytometry. Representative experiments are presented by dot plots in d. Data as in D were quantified as the percentage of cells expressing IL‐10 above background € and as MFI of IL‐10 in CD4+CD25+CD127‐ gated cells (f), from seven independent experiments (mean ± SD).

Given the effects of spEVs on T‐cell functions, potentially driving immune tolerance, we next investigated whether spEVs promote the differentiation of Tregs. Tregs comprise a specialized subset of T‐cells that are characterized by the expression of the transcription factor Foxp3, which is crucial for their development and immune suppressive functions (Rudensky, 2011). Naturally occurring Tregs in human peripheral blood have been defined as CD4+CD25+CD127low T‐cells (Yu et al., 2012), and also as CD4+CD25+Foxp3+ T‐cells (Zheng & Rudensky, 2007). Therefore, we first tested the effect of spEVs on the occurrence of Foxp3 expressing CD3+CD4+CD25+CD127‐ T‐cells in PBMCs. Hereto, PBMCs were cultured for 3 days in the presence or absence of spEVs (Figure S7). At these conditions, the contribution of CD3+CD4+CD25+CD127‐ T‐cells did not increase significantly, but the expression of Foxp3 increased 1.7 ± 0.3 fold. To investigate direct effects of spEVs on T‐cells during their activation, we then continued by studying isolated pan T‐cells (Figure 7a–c). Hereto, isolated pan T‐cells were activated with anti‐CD3/CD28 coated Dynabeads either in the absence or presence of spEVs. In the absence of spEVs, 43 ± 7% of the activated CD3+CD4+CD25+CD127‐ T‐cells expressed Foxp3 above background. In the presence of spEVs, however, this number almost doubled to 74 ± 2%, while the average expression of Foxp3 in CD3+CD4+CD25+CD127‐ T‐cells, as determined by MFI, increased 4.8 ± 1.4 fold (Figure 7b–c). It should be noted that Tregs versus activated nonsuppressive T‐cells are defined by the concentration of Foxp3 rather than by its absolute absence or presence (Wang et al., 2007). Another hallmark of Treg differentiation is their increase in IL‐10 production. Using a flow cytometry approach, we indeed detected an increase in intracellular IL‐10 in CD3+CD4+CD25+CD127‐ T‐cells (Figure 7d, e, and Figure S8). Collectively, these data demonstrate that spEVs strongly primed CD4+ T‐cells to differentiate into Tregs when activated, independently from APC.

### 3.5 spEVs interfere with T‐cell function through A2AR signalling

**FIGURE 8:**
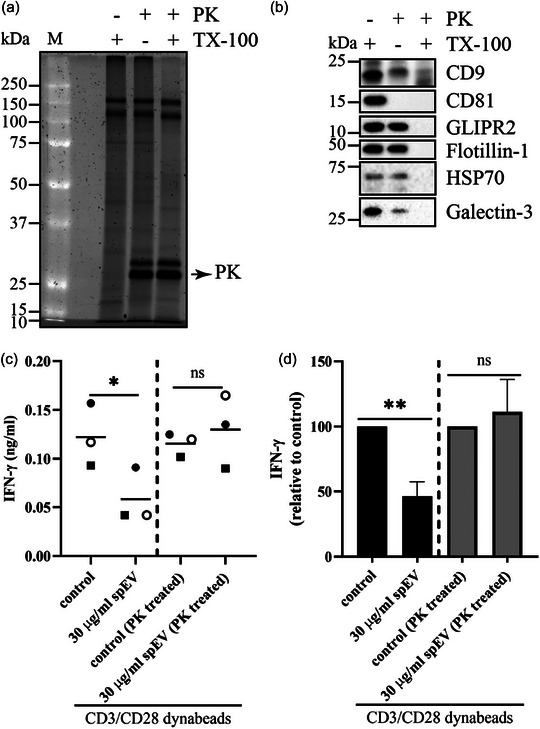
spEV surface proteins are instrumental in modulating T‐cell activation. (a, b) Isolated spEVs were treated with proteinase K (PK) in the presence or absence of triton X‐100 (TX‐100) as indicated, and then analysed by SDS‐PAGE, followed by total protein staining (A), or by immunoblotting for the presence of CD9, CD81, GLIPR2, Flotillin‐1, HSP70, and Galectin‐3. (b). Molecular weight markers are indicated on the left in kDa. In the absence of TX‐100, only surface exposed proteins, but not intraluminal proteins, can be degraded by PK. (C and D), Isolated pan T‐cells were stimulated for 16 h with anti‐CD3/CD28 Dynabeads, either in the absence or presence of non‐treated or PK treated spEVs, as indicated. Samples marked by “control (PK treated)” were incubated with a PK treated vehicle that was prepared in parallel with the PK treated spEVs but lacked spEVs. IFN‐γ in cell culture media was determined by ELISA and plotted as ng/mL **(c)**, or relative to control **(d)** (mean ± SD, from three independent experiments).

Next, we searched for molecular mechanisms by which spEVs can interfere with T‐cell function. spEVs can be expected to rely on proteins on their surface that specifically bind to recipient T‐cells. To study the potential role of membrane proteins, spEVs were incubated with proteinase K to strip surface‐exposed proteins (Figures 8a, b). The majority of spEV proteins were resistant to this treatment, consistent with protection of their cytoplasmic content by the spEVs membrane. Indeed, most proteins became susceptible to degradation by proteinase K only in the additional presence of the membrane‐disrupting detergent TX‐100. Surface‐exposed CD81 epitopes were fully eliminated by proteinase K already in the absence of TX‐100, while the CD9 epitopes were only partly degraded at these conditions. Cytosolic proteins, including GLIPR2, Flotillin‐1, Annexin A1, and HSP70, were fully protected by the membrane and therefore not affected by the protease in the absence of TX‐100. Galectin‐3 was partially susceptible to proteinase K digestion in the absence of TX‐100, consistent with its presence both in the cytoplasmic lumen and at the exoplasmic surface of EV membranes (Jones et al., 2010; Popa et al., 2018). The effect of spEVs on T‐cell function, as determined here by their interference with IFN‐γ production in response to T‐cell activation, was fully abrogated by the proteinase K treatment (Figures 8c, d). These data indicate that spEVs require surface‐exposed membrane proteins to modulate T‐cell function.

**FIGURE 9:**
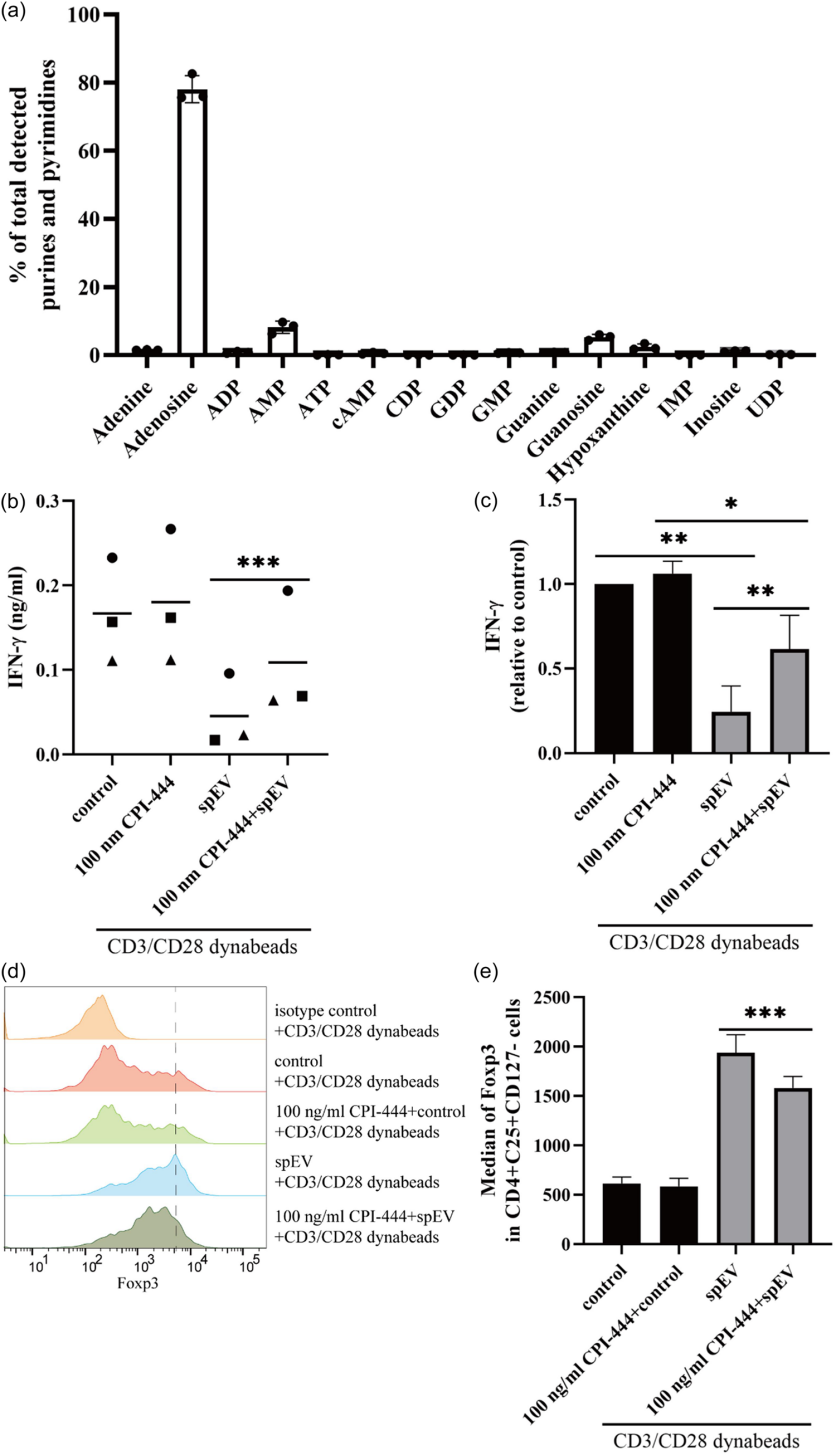
spEVs carry adenosine and regulate T‐cell function via A2AR. (a) spEVs were analysed for the presence of purines and pyrimidines using mass spectrometry. Adenosine was highly enriched in palleted spEVs relative to other purines (mean ± SD, from three independent experiments). (b, c) Isolated pan T‐cells were activated for 16 h by anti‐CD3/CD28 Dynabeads, either in the absence or presence of only 100 nM CPI‐444, only 60 μg/mL spEVs, or both CPI‐444 and spEVs, as indicated. IFN‐γ in cell culture media was then determined by ELISA and plotted as ng/mL (b, distinct symbols indicate independent experiments), or relative to control (C, mean ± SD, *n* = 3 independent experiments). (d, e) Isolated Pan T‐cells were incubated for 2 h in the absence or presence of 100 nM CPI‐444, after which 60 μg/mL spEVs was added when indicated. Subsequentially, the pan T cells were stimulated with CD3/CD28 Dynabeads, when indicated. After 4 days, the cells were analysed by flow cytometry for Foxp3 expression in CD3+CD4+CD25+CD127‐ cells. (d) Representative histograms indicating Foxp3 labelling in CD4+CD25+CD127‐ gated cells. The top graph displays labelling with an isotype control antibody. (e), MFI (mean ± SD, *n* = 4 independent experiments of data as in d.

Given that Foxp3 expression and Treg differentiation can be driven by adenosine A2A receptor (A2AR) signalling (Ohta et al., 2012; Zheng & Rudensky, 2007), we hypothesized that spEVs may induce Treg development by transferring adenosine, the endogenous activating ligand of A2AR, to T‐cells. Consistent with this idea, analysis of the metabolites in spEVs, revealed that adenosine is highly enriched relative to other purine and pyrimidine metabolites (Figure 9a). To demonstrate a direct role of A2AR in spEV signalling, we next tested the effects of CPI‐444, a selective antagonist of A2AR that has been widely used to investigate the role of A2AR signalling in T cell differentiation and Treg development (Ohta et al., 2012; Willingham et al., 2018). Isolated pan T‐cells were activated for 16 h by CD3/CD28 Dynabeads, either in the absence or presence of CPI‐444 and/or spEVs. In these experiments, expression of Foxp3 and IFN‐γ was taken as a readout for interference with T cell differentiation. CPI‐444 reduced the inhibitory effect of spEVs on IFN‐γ production by T‐cells in a concentration dependent manner (Figures 9b, c, and S9) and also reduced the spEV induced expression of Foxp3 (Figure 9d–e), indicating that spEVs signal to T cells with the aid of A2AR. Based on our collective data, one possible hypothesis, requiring further investigation, is that endocytosed spEVs may release their adenosine content into the lumen of endosomes, thus allowing endosome localized A2AR receptor signalling in spEVs targeted T‐cells.

## 4 DISCUSSION

In this study, we observed that isolated spEVs interact with and are endocytosed by both monocytes and T‐cells, and explored the effects of spEVs on the differentiation of activated T‐cells. We found that spEVs have a direct, potent and sustained effect on T‐cell activation, as measured by a reduction in CD25 surface expression, interference with T‐cell proliferation, and reduced expression of IFN‐γ, TNF, and IL‐2. In contrast, spEVs increased the expression of IL‐10 and upregulated the development of Foxp3 expressing CD4+CD25+CD127‐ T cells. All these hallmarks are consistent with T‐cell differentiation into Tregs. In our study, the spEVs stimulated differentiation of CD4+ T‐cells towards the Treg phenotype also occurred when the cells were activated by anti‐CD3/CD28 Dynabeads in the absence of antigen‐presenting cells, indicating a role for direct interaction of spEVs with T‐cells. A direct effect was confirmed by the MLR experiment in which T‐cells were first incubated with spEVs, then washed to remove excess spEVs, and subsequently activated by APCs present in allogeneic PBMCs (Figure 2c). In this study we did not explore whether spEVs also interfere with T‐cell development in an indirect way through interactions with monocytes or other APCs. Our study is consistent with other studies that demonstrated induction of in vitro expansion of Tregs by unfractionated seminal plasma, in the absence of APCs (Meuleman et al., 2015; Robertson et al., 2009). The physiological relevance of our in vitro findings for a role of spEVs in modulating T‐cells in vivo remains to be established. In order for spEVs to contact T‐cells after vaginal intercourse, spEVs must first cross the epithelial barrier of the genital tract. The ectocervix and vagina are covered by a stratified squamous non‐keratinized epithelium, with many T‐cells situated within the vaginal epithelium and in the superficial lamina propria (Doncel et al., 2014; Miller & Shattock, 2003). Light microscopic and electron microscopic analysis of the vaginal mucosa demonstrated that lymphocytes can migrate through the lamina propria and the basement membrane, penetrating the space between epithelial cells (Roig de & Burgos, 1968; Saba et al., 2010). The question whether T cells in the lower FRT can interact with spEVs after vaginal intercourse cannot be answered yet. However, the fact that T cells in the cervix and in cervical explants are susceptible to infection by human immunodeficiency virus (HIV) (Doncel et al., 2014; Miller & Shattock, 2003), suggests that they can. HIV is an enveloped virus with a diameter of ∼100 nm, comparable to that of spEVs. One possibility for spEVs to get in direct contact with T cells is through vaginal epithelial tears. Vaginal epithelial tears have been detected in >60% of women following consensual sexual intercourse (Norvell et al., 1984). Evidence from human ex vivo systems suggests that when microscopic or macroscopic defects in the epithelium occur, HIV has direct access to susceptible T cells (Saba et al., 2010). These data suggest that T cells present within in the vaginal or ectocervix epithelium may, similar to HIV, also be accessible to spEV following sexual intercourse. The highest concentration of spEVs used in our study was 60 μg/mL, which is nearly 7‐fold less as compared to the ∼0.4 mg/mL concentration of spEVs in seminal plasma, based on our calculations on the recovery of isolated spEVs and their originating volume of seminal plasma. Considering that semen is diluted upon deposition in the FRT, the in vitro dose‐response effects of isolated spEVs on immune cells that we report here can be expected to match that of in situ conditions. CD3+ T‐cells comprise a major cell population in the cervix (Poppe et al., 1998), which increases substantially within both the epithelium and stroma after exposure to seminal plasma (Sharkey et al., 2012). Our findings are consistent with the observation that seminal plasma stimulates the recruitment of Tregs into the FRT (Robertson et al., 2009). Our data are also in agreement with observations that exposure of PBMCs to seminal plasma increased IL‐10 mRNA expression in PBMCs and Foxp3 mRNA expression in T‐cells (Ohta et al., 2012), and inhibited T‐cell functions (Selva et al., 2017). Our data indicate how spEVs may help to prevent immune‐mediated recognition and destruction of allogeneic spermatozoa within the female FRT (Skibinski et al., 1992).

Several studies have implicated spEVs to interfere with the capacity of antigen‐presenting cells to activate antigen‐specific memory CD8+ T‐cells (Vojtech et al., 2019), and to stimulate the differentiation of monocytes into tolerogenic DCs (Remes Lenicov et al., 2012; Yasuda et al., 2020). Consistent with this idea, we found that Alexa488‐spEVs were recruited, apart from T‐cells, predominantly by CD14+ monocytes (Figures 3 and 4). In our current study, we did not pursue the biological implications of the recruitment spEVs by monocytes. One likely scenario is that immune regulatory cells are recruited from the cervical and uterine mucosal immune system in response to prostaglandin E (PGE), TGF‐β, and other cytokines, which in the majority are produced and released into seminal plasma by the seminal vesicle glands (Sharkey et al., 2007; Yasuda et al., 2020), and that spEVs from the prostate are subsequently required for immune cell tolerization. Soluble mediators from the seminal vesicle glands may in this way prime immune tolerance of the FRT in concert with prostate‐derived spEVs. Importantly though, an alternative scenario in which seminal vesicle glands also serve an important role in the production of immune tolerizing spEVs cannot be excluded. Indeed, the prostate is not the only source of spEVs. Because we use seminal plasma from vasectomized men, we can exclude contributions from the testis and the epididymis, but not from seminal vesicle glands. EVs have indeed been found in the aspirated fluid from operationally dissected seminal vesicle glands (Sahlén et al., 2010). Moreover, both 50 and 100 nm spEVs subpopulations that we have isolated from seminal plasma contained, in addition to the many prostate‐specific proteins, also some proteins that are specifically expressed in seminal vesicle glands (Zhang et al., 2020a). A role for seminal vesicle gland‐derived spEVs may concur with the observation that while lymphocytes in the draining lymph nodes of the uterus of mice are activated in response to mating, this was not observed after mating with male mice in which the seminal vesicle glands had been surgically removed (Johansson et al., 2004; Yasuda et al., 2020). The latter experiments are, however, not indicative for the origin of immune modulatory spEVs since also non‐spEV associated immune‐regulators, including PGE, TGF‐β, and other cytokines, are produced and released into seminal plasma by the seminal vesicle glands (Miller & Shattock, 2003; Yasuda et al., 2020).

We and others have previously reported on other activities of spEVs (Aalberts et al., 2013a; Aalberts et al 2013b; Arienti et al., 1997; Ronquist, 2012). Isolated spEVs can also bind to sperm cells, and in vitro seem to stimulate their motility and regulate the timing of capacitation, as well as the induction of the acrosome reaction. Further work is required to determine whether spEVs that act on sperm cell functions are distinct from those that act on immune cells. In our previous studies, we isolated two subpopulations of spEVs from the seminal plasma of vasectomized man, that are differing in size (50 nm vs. 100 nm), as well as in protein and lipid compositions (Aalberts et al., 2012; Brouwers et al., 2013; Zhang et al., 2020a). Their separation was based on velocity sucrose gradient centrifugation. However, given that sucrose density gradients are highly hypertonic and may have detrimental osmotic effects on EV functional activities, we switched to iohexol density gradients in the current study. Iohexol has a much lower osmolarity compared to sucrose, and previously we have successfully applied iohexol density gradient centrifugation to isolate EVs from blood and cell culture media (Zhang et al., 2020 In our current protocol (Figures 1 and S1), we have modified and optimized the procedure of iohexol density gradient centrifugation for spEVs isolation. While sucrose density gradients could separate the two populations of 50 and 100 nm spEVs, iohexol density gradients could not. In both iohexol and sucrose density gradients, 50 and 100 nm spEVs eventually migrated to a shared equilibrium density after prolonged centrifugation. In comparison, sucrose has a much higher viscosity than iohexol, thus allowing separation of 50 nm from 100 nm spEVs during sucrose gradient centrifugation, by making use of their distinct velocities by which they reach their shared equilibrium density (Aalberts et al., 2012). In a previous study, we identified 1558 proteins in spEVs by using quantitative Liquid Chromatography‐Mass spectrometry (Zhang et al., 2020a). Fifty four percent of these proteins were shared by 50 nm and 100 nm spEVs, with the remainder exclusively detected in either 50 or 100 nm spEVs, suggesting that these spEVs subpopulations may serve distinct functions. Gene ontology (GO) enrichment analysis suggested that the majorities of both spEVs subpopulations originate from the prostate but with distinct biogenesis pathways. Using GO enrichment analysis we have identified 30 proteins in isolated spEVs that could be annotated for immune regulation (Zhang et al., 2020a).

The A2AR plays a crucial role in modulating the immune system, maintaining immune homeostasis (Ohta & Sitkovsky, 2001). For example, A2AR‐deficient mice display reduced numbers and impaired suppressive function of Tregs, highlighting the essential role of A2AR in Treg homeostasis (Ohta et al., 2012). Activation of A2AR by its endogenous ligand adenosine leads to the downstream activation of the cAMP signalling pathway (Ohta et al., 2006). In the context of Tregs, A2AR‐induced cAMP signalling has been shown to positively regulate Foxp3 expression, favouring Treg development (Ma et al., 2017). Specifically, A2AR signalling enhances the suppressive capacity of Tregs by promoting the secretion of anti‐inflammatory cytokines such as IL‐10 and TGF‐β, and inhibiting the production of pro‐inflammatory cytokines, including IFN‐γ, TNF, and IL‐2 (Zarek et al., 2008). We have demonstrated here that spEVs can induce such a cytokine fingerprint, consistent with the role of adenosine mediated signalling. A2AR could not be detected in our isolated spEVs (Zhang et al., 2020a), and it is thus likely that spEVs function by transferring adenosine rather than A2AR to T‐cells. Others have demonstrated that EVs from cancer cells can also suppress T cells through adenosine signalling via A2AR, but in that case, exoplasmic adenosine seems to be generated by cancer EVs from extracellular ATP by the subsequent action of CD39, for the convesion of extracellular ATP or ADP to 5’‐AMP, and CD73 (5’‐nucleotidase) for the conversion of 5’‐AMP to adenosine (Morandi et al. 2018; Clayton et al. 2011; (Wang et al., 2021). We detected CD73 but not CD39 in our isolated spEVs (Zhang et al., 2020a), and in our current in vitro experiments extracellular ATP was not present during in vitro T‐cell activation, making it unlikely that spEVs generated adenosine via this pathway. Instead, we observed a relatively high concentration of adenosine in isolated spEVs (Figure 9a). While mechanisms responsible for spEV effects on T cell differentiation still need to be elucidated, we demonstrate that spEVs are endocytosed by T‐cells and targeted to acidified compartments (Figure 4b–d). We propose that adenosine may leak from endocytosed spEVs into the endosomal lumen, potentially allowing binding to endosomal A2AR. Although endocytosis of A2AR is triggered to some extend by adenosine binding (Brand et al., 2008), also non‐activated A2ARs can undergo ligand‐independent endocytosis, and depending on the internalization pathway, endocytosed receptors may provide a scaffold for active signalling components (DeWire et al., 2007; Klaasse et al., 2008). Whether and how adenosine is leaked out from spEVs into the endosomal lumen is not clear but either adenosine transporters or destabilization of the spEV membrane by the endosomal/lysosomal environment may contribute to the process.

Tregs are beneficial by suppressing immune responses towards allogeneic sperm, but in the male may also limit antitumor immunity in case of prostate cancer. More specifically, A2AR drives CD8+ T cell tolerance in the tumour microenvironment (Cekic & Linden, 2014). The here reported immunomodulatory functions of spEVs thus have significant implications in various physiological and pathological processes, Understanding the role of spEVs in A2AR signalling in T‐cells may lead to innovative therapeutic approaches with regard to infertility and to increase the success rate of embryo transplantation after in vitro fertilization, but also in relation to prostate cancer. Further investigations are therefore warranted to fully understand the mechanisms and functional consequences of spEV‐mediated A2AR signalling and to explore potential clinical applications. In conclusion, we here demonstrate that spEVs have immune‐tolerizing capacities in vitro, and in semen may function in tandem with soluble immune regulatory components to promote an immune tolerogenic environment within the FRT to paternal alloantigenic semen and possibly to prevent allogeneic foetal rejection. Potential regulatory capacities of spEVs towards other immune cell types remain largely unexplored, and additional studies, both in vitro and in vivo, are required to fully understand how spEVs regulate adaptive immunity.

## AUTHOR CONTRIBUTIONS

Willem Stoorvogel conceived the study. Willem Stoorvogel, Xiaogang Zhang, Marianne Boes, Richard Wubbolts, Celia R. Berkers, and Esther A. Zaal designed experiments. Xiaogang Zhang, Patrick F. Greve, and Thi Ngoc Minh Tran performed experiments. Ayşe Y. Demir organized seminal plasma donation and quality control. Xiaogang Zhang and Willem Stoorvogel wrote the manuscript, and all authors contributed to revision and editing of the manuscript.

## DECLARATION OF INTERESTS

The authors declare no competing interests.

## Supporting information

Supplementary Information

Supplementary Information

## References

[jev212457-bib-0030] Aalberts, M. , Stout, T. A. , & Stoorvogel, W. (2013a). Prostasomes: extracellular vesicles from the prostate. Reproduction (Cambridge, England), 147(1), R1–R14. 24149515 10.1530/REP-13-0358

[jev212457-bib-0036] Aalberts, M. , Sostaric, E. , Wubbolts, R. , Wauben, M. W. , Nolte‐’t Hoen, E. N. , Gadella, B. M. , Stout, T. A. , & Stoorvogel, W. (2013b). Spermatozoa recruit prostasomes in response to capacitation induction. Biochimica et Biophysica Acta, 1834(11), 2326–2335. 22940639 10.1016/j.bbapap.2012.08.008

[jev212457-bib-0040] Aalberts, M. , van Dissel‐Emiliani, F. M. , van Adrichem, N. P. , van Wijnen, M. , Wauben, M. H. , Stout, T. A. , & Stoorvogel, W. (2012). Identification of distinct populations of prostasomes that differentially express prostate stem cell antigen, annexin A1, and GLIPR2 in humans. Biology of Reproduction, 86(3), 82. 22133690 10.1095/biolreprod.111.095760

[jev212457-bib-0062] Arienti, G. , Carlini, E. , Verdacchi, R. , & Palmerini, C. A. (1997). Transfer of aminopeptidase activity from prostasomes to sperm. Biochimica et Biophysica Acta, 1336(2), 269–274. 9305799 10.1016/s0304-4165(97)00036-6

[jev212457-bib-0071] Brand, F. , Klutz, A. M. , Jacobson, K. A. , Fredholm, B. B. , & Schulte, G. (2008). Adenosine A(2A) receptor dynamics studied with the novel fluorescent agonist Alexa488‐APEC. European Journal of Pharmacology, 590(1‐3), 36–42. 18603240 10.1016/j.ejphar.2008.05.036 PMC2765816

[jev212457-bib-0063] Brouwers, J. F. , Aalberts, M. , Jansen, J. W. , van Niel, G. , Wauben, M. H. , Stout, T. A. , Helms, J. B. , & Stoorvogel, W. (2013). Distinct lipid compositions of two types of human prostasomes. Proteomics, 13(10‐11), 1660–1666. 23404715 10.1002/pmic.201200348

[jev212457-bib-0074] Cekic, C. , & Linden, J. (2014). Adenosine A2A receptors intrinsically regulate CD8+ T cells in the tumor microenvironment. Cancer Research, 74(24), 7239–7249. 25341542 10.1158/0008-5472.CAN-13-3581 PMC4459794

[jev212457-bib-0001] Chen, C. , Song, X. , Wei, W. , Zhong, H. , Dai, J. , Lan, Z. , Li, F. , Yu, X. , Feng, Q. , Wang, Z. , Xie, H. , Chen, X. , Zeng, C. , Wen, B. , Zeng, L. , Du, H. , Tang, H. , Xu, C. , Xia, Y. , … Jia, H. (2017). The microbiota continuum along the female reproductive tract and its relation to uterine‐related diseases. Nature Communications, 8(1), 875. 10.1038/s41467-017-00901-0 PMC5645390 29042534

[jev212457-bib-0070] Clayton, A. , Al‐Taei, S. , Webber, J. , Mason, M. D. , & Tabi, Z. (2011). Cancer exosomes express CD39 and CD73, which suppress T cells through adenosine production. Journal of Immunology (Baltimore, Md. : 1950), 187(2), 676–683. 21677139 10.4049/jimmunol.1003884

[jev212457-bib-0017] Crawford, G. , Ray, A. , Gudi, A. , Shah, A. , & Homburg, R. (2015). The role of seminal plasma for improved outcomes during in vitro fertilization treatment: review of the literature and meta‐analysis. Human Reproduction Update, 21(2), 275–284. 25281684 10.1093/humupd/dmu052

[jev212457-bib-0007] De, M. , Choudhuri, R. , & Wood, G. W. (1991). Determination of the number and distribution of macrophages, lymphocytes, and granulocytes in the mouse uterus from mating through implantation. Journal of Leukocyte Biology, 50(3), 252–262. 1856596 10.1002/jlb.50.3.252

[jev212457-bib-0072] DeWire, S. M. , Ahn, S. , Lefkowitz, R. J. , & Shenoy, S. K. (2007). Beta‐arrestins and cell signaling. Annual Review of Physiology, 69, 483–510. 10.1146/annurev.physiol.69.022405.154749 17305471

[jev212457-bib-0053] Doncel, G. F. , Anderson, S. , & Zalenskaya, I. (2014). Role of semen in modulating the female genital tract microenvironment–implications for HIV transmission. American Journal of Reproductive Immunology (New York, N.Y. : 1989), 71(6), 564–574. 24702729 10.1111/aji.12231

[jev212457-bib-0035] Höög, J. L. , & Lötvall, J. (2015). Diversity of extracellular vesicles in human ejaculates revealed by cryo‐electron microscopy. Journal of Extracellular Vesicles, 4, 28680. 26563734 10.3402/jev.v4.28680 PMC4643196

[jev212457-bib-0004] Hansen, P. J. (2011). The immunology of early pregnancy in farm animals. Reproduction in Domestic Animals = Zuchthygiene, 46, (Suppl 3), 18–30. 10.1111/j.1439-0531.2011.01850.x 21854458

[jev212457-bib-0023] Hancock, R. J. , & Faruki, S. (1986). Assessment of immune responses to H‐Y antigen in naturally inseminated and sperm‐injected mice using cell‐mediated cytotoxicity assays. Journal of Reproductive Immunology, 9(3), 187–194. 3806527 10.1016/0165-0378(86)90012-4

[jev212457-bib-0006] Huang, N. , Chi, H. , & Qiao, J. (2020). Role of regulatory T cells in regulating fetal‐maternal immune tolerance in healthy pregnancies and reproductive diseases. Frontiers in Immunology, 11, 1023. 32676072 10.3389/fimmu.2020.01023 PMC7333773

[jev212457-bib-0014] Introini, A. , Boström, S. , Bradley, F. , Gibbs, A. , Glaessgen, A. , Tjernlund, A. , & Broliden, K. (2017). Seminal plasma induces inflammation and enhances HIV‐1 replication in human cervical tissue explants. PLoS Pathogens, 13(5), e1006402. 28542587 10.1371/journal.ppat.1006402 PMC5453613

[jev212457-bib-0021] Johansson, M. , Bromfield, J. J. , Jasper, M. J. , & Robertson, S. A. (2004). Semen activates the female immune response during early pregnancy in mice. Immunology, 112(2), 290–300. 15147572 10.1111/j.1365-2567.2004.01876.x PMC1782486

[jev212457-bib-0048] Jones, J. L. , Saraswati, S. , Block, A. S. , Lichti, C. F. , Mahadevan, M. , & Diekman, A. B. (2010). Galectin‐3 is associated with prostasomes in human semen. Glycoconjugate Journal, 27(2), 227–236. 19830550 10.1007/s10719-009-9262-9 PMC3261635

[jev212457-bib-0027] Kelly, R. W. , Holland, P. , Skibinski, G. , Harrison, C. , McMillan, L. , Hargreave, T. , & James, K. (1991). Extracellular organelles (prostasomes) are immunosuppressive components of human semen. Clinical and Experimental Immunology, 86(3), 550–556. 1747961 10.1111/j.1365-2249.1991.tb02968.x PMC1554200

[jev212457-bib-0026] Kitamura, M. , Namiki, M. , Matsumiya, K. , Tanaka, K. , Matsumoto, M. , Hara, T. , Kiyohara, H. , Okabe, M. , Okuyama, A. , & Seya, T. (1995). Membrane cofactor protein (CD46) in seminal plasma is a prostasome‐bound form with complement regulatory activity and measles virus neutralizing activity. Immunology, 84(4), 626–632. 7790037 PMC1415160

[jev212457-bib-0073] Klaasse, E. C. , Ijzerman, A. P. , de Grip, W. J. , & Beukers, M. W. (2008). Internalization and desensitization of adenosine receptors. Purinergic Signalling, 4(1), 21–37. 18368531 10.1007/s11302-007-9086-7 PMC2245999

[jev212457-bib-0022] Lengerova, A. , & Vojtiskova, M. (1963). Prolonged survival of syngeneic male skin grafts in parous C57B1 mice. Folia Biologica, 9, 72–74. 13929620

[jev212457-bib-0066] Ma, S. R. , Deng, W. W. , Liu, J. F. , Mao, L. , Yu, G. T. , Bu, L. L. , Kulkarni, A. B. , Zhang, W. F. , & Sun, Z. J. (2017). Blockade of adenosine A2A receptor enhances CD8^+^ T cells response and decreases regulatory T cells in head and neck squamous cell carcinoma. Molecular Cancer, 16(1), 99. 28592285 10.1186/s12943-017-0665-0 PMC5461710

[jev212457-bib-0042] Majzoub, R. N. , Wonder, E. , Ewert, K. K. , Kotamraju, V. R. , Teesalu, T. , & Safinya, C. R. (2016). Rab11 and lysotracker markers reveal correlation between endosomal pathways and transfection efficiency of surface‐functionalized cationic liposome‐DNA nanoparticles. The Journal of Physical Chemistry. B, 120(26), 6439–6453. 27203598 10.1021/acs.jpcb.6b04441 PMC4936928

[jev212457-bib-0008] Marlin, R. , Nugeyre, M. T. , Tchitchek, N. , Parenti, M. , Lefebvre, C. , Hocini, H. , Benjelloun, F. , Cannou, C. , Nozza, S. , Dereuddre‐Bosquet, N. , Levy, Y. , Barré‐Sinoussi, F. , Scarlatti, G. , Le Grand, R. , & Menu, E. (2019). Seminal plasma exposures strengthen vaccine responses in the female reproductive tract mucosae. Frontiers in Immunology, 10, 430. 30915079 10.3389/fimmu.2019.00430 PMC6423065

[jev212457-bib-0051] Meuleman, T. , Snaterse, G. , van Beelen, E. , Anholts, J. D. , Pilgram, G. S. , van der Westerlaken, L. A. , Eikmans, M. , & Claas, F. H. (2015). The immunomodulating effect of seminal plasma on T cells. Journal of Reproductive Immunology, 110, 109–116. 25799173 10.1016/j.jri.2015.01.012

[jev212457-bib-0052] Miller, C. J. , & Shattock, R. J. (2003). Target cells in vaginal HIV transmission. Microbes and Infection, 5(1), 59–67. 12593974 10.1016/s1286-4579(02)00056-4

[jev212457-bib-0069] Morandi, F. , Marimpietri, D. , Horenstein, A. L. , Bolzoni, M. , Toscani, D. , Costa, F. , Castella, B. , Faini, A. C. , Massaia, M. , Pistoia, V. , Giuliani, N. , & Malavasi, F. (2018). Microvesicles released from multiple myeloma cells are equipped with ectoenzymes belonging to canonical and non‐canonical adenosinergic pathways and produce adenosine from ATP and NAD. Oncoimmunology, 7(8), e1458809. 30221054 10.1080/2162402X.2018.1458809 PMC6136872

[jev212457-bib-0002] Nederlof, I. , Meuleman, T. , van der Hoorn, M. L. P. , Claas, F. H. J. , & Eikmans, M. (2017). The seed to success: The role of seminal plasma in pregnancy. Journal of Reproductive Immunology, 123, 24–28. 28886486 10.1016/j.jri.2017.08.008

[jev212457-bib-0056] Norvell, M. K. , Benrubi, G. I. , & Thompson, R. J. (1984). Investigation of microtrauma after sexual intercourse. The Journal of Reproductive Medicine, 29(4), 269–271. 6716372

[jev212457-bib-0049] Ohta, A. , Kini, R. , Ohta, A. , Subramanian, M. , Madasu, M. , & Sitkovsky, M. (2012). The development and immunosuppressive functions of CD4(+) CD25(+) FoxP3(+) regulatory T cells are under influence of the adenosine‐A2A adenosine receptor pathway. Frontiers in Immunology, 3, 190. 22783261 10.3389/fimmu.2012.00190 PMC3389649

[jev212457-bib-0064] Ohta, A. , & Sitkovsky, M. (2001). Role of G‐protein‐coupled adenosine receptors in downregulation of inflammation and protection from tissue damage. Nature, 414(6866), 916–920. 11780065 10.1038/414916a

[jev212457-bib-0065] Ohta, A. , Gorelik, E. , Prasad, S. J. , Ronchese, F. , Lukashev, D. , Wong, M. K. , Huang, X. , Caldwell, S. , Liu, K. , Smith, P. , Chen, J. F. , Jackson, E. K. , Apasov, S. , Abrams, S. , & Sitkovsky, M. (2006). A2A adenosine receptor protects tumors from antitumor T cells. Proceedings of the National Academy of Sciences of the United States of America, 103(35), 13132–13137. 16916931 10.1073/pnas.0605251103 PMC1559765

[jev212457-bib-0047] Popa, S. J. , Stewart, S. E. , & Moreau, K. (2018). Unconventional secretion of annexins and galectins. Seminars in Cell & Developmental Biology, 83, 42–50. 29501720 10.1016/j.semcdb.2018.02.022 PMC6565930

[jev212457-bib-0057] Poppe, W. A. , Drijkoningen, M. , Ide, P. S. , Lauweryns, J. M. , & Van Assche, F. A. (1998). Lymphocytes and dendritic cells in the normal uterine cervix. An immunohistochemical study. European Journal of Obstetrics, Gynecology, and Reproductive Biology, 81(2), 277–282. 9989877 10.1016/s0301-2115(98)00202-4

[jev212457-bib-0039] Reiner, A. T. , Witwer, K. W. , van Balkom, B. W. M. , de Beer, J. , Brodie, C. , Corteling, R. L. , Gabrielsson, S. , Gimona, M. , Ibrahim, A. G. , de Kleijn, D. , Lai, C. P. , Lötvall, J. , Del Portillo, H. A. , Reischl, I. G. , Riazifar, M. , Salomon, C. , Tahara, H. , Toh, W. S. , Wauben, M. H. M. , … Lim, S. K. (2017). Concise review: Developing best‐practice models for the therapeutic use of extracellular vesicles. Stem Cells Translational Medicine, 6(8), 1730–1739. 28714557 10.1002/sctm.17-0055 PMC5689784

[jev212457-bib-0019] Remes Lenicov, F. , Rodriguez Rodrigues, C. , Sabatté, J. , Cabrini, M. , Jancic, C. , Ostrowski, M. , Merlotti, A. , Gonzalez, H. , Alonso, A. , Pasqualini, R. A. , Davio, C. , Geffner, J. , & Ceballos, A. (2012). Semen promotes the differentiation of tolerogenic dendritic cells. Journal of Immunology, 189(10), 4777–4786. 10.4049/jimmunol.1202089 23066152

[jev212457-bib-0005] Robertson, S. A. , Care, A. S. , & Moldenhauer, L. M. (2018). Regulatory T cells in embryo implantation and the immune response to pregnancy. The Journal of Clinical Investigation, 128(10), 4224–4235. 30272581 10.1172/JCI122182 PMC6159994

[jev212457-bib-0009] Robertson, S. A. , Mau, V. J. , Tremellen, K. P. , & Seamark, R. F. (1996). Role of high molecular weight seminal vesicle proteins in eliciting the uterine inflammatory response to semen in mice. Journal of Reproduction and Fertility, 107(2), 265–277. 8882294 10.1530/jrf.0.1070265

[jev212457-bib-0013] Robertson, S. A. , Prins, J. R. , Sharkey, D. J. , & Moldenhauer, L. M. (2013). Seminal fluid and the generation of regulatory T cells for embryo implantation. American Journal of Reproductive Immunology, 69(4), 315–330. 23480148 10.1111/aji.12107

[jev212457-bib-0016] Robertson, S. A. (2005). Seminal plasma and male factor signalling in the female reproductive tract. Cell and Tissue Research, 322(1), 43–52. 15909166 10.1007/s00441-005-1127-3

[jev212457-bib-0020] Robertson, S. A. , Guerin, L. R. , Bromfield, J. J. , Branson, K. M. , Ahlström, A. C. , & Care, A. S. (2009). Seminal fluid drives expansion of the CD4+CD25+ T regulatory cell pool and induces tolerance to paternal alloantigens in mice. Biology of Reproduction, 80(5), 1036–1045. 19164169 10.1095/biolreprod.108.074658 PMC2849830

[jev212457-bib-0038] Roberts‐Dalton, H. D. , Cocks, A. , Falcon‐Perez, J. M. , Sayers, E. J. , Webber, J. P. , Watson, P. , Clayton, A. , & Jones, A. T. (2017). Fluorescence labelling of extracellular vesicles using a novel thiol‐based strategy for quantitative analysis of cellular delivery and intracellular traffic. Nanoscale, 9(36), 13693–13706. 28880029 10.1039/c7nr04128d

[jev212457-bib-0055] de Roig, V.‐L. , & Burgos, M. H. (1968). Migration of lymphocytes in the normal human vagina. American Journal of Obstetrics and Gynecology, 102(8), 1094–1101. 5699763 10.1016/0002-9378(68)90398-0

[jev212457-bib-0029] Ronquist, G. , & Brody, I. (1985). The prostasome: its secretion and function in man. Biochimica et Biophysica Acta, 822(2), 203–218. 2992593 10.1016/0304-4157(85)90008-5

[jev212457-bib-0061] Ronquist, G. (2012). Prostasomes are mediators of intercellular communication: From basic research to clinical implications. Journal of Internal Medicine, 271(4), 400–413. 22112042 10.1111/j.1365-2796.2011.02487.x

[jev212457-bib-0025] Rooney, I. A. , Atkinson, J. P. , Krul, E. S. , Schonfeld, G. , Polakoski, K. , Saffitz, J. E. , & Morgan, B. P. (1993). Physiologic relevance of the membrane attack complex inhibitory protein CD59 in human seminal plasma: CD59 is present on extracellular organelles (prostasomes), binds cell membranes, and inhibits complement‐mediated lysis. The Journal of Experimental Medicine, 177(5), 1409–1420. 7683035 10.1084/jem.177.5.1409 PMC2191001

[jev212457-bib-0043] Rudensky, A. Y. (2011). Regulatory T cells and Foxp3. Immunological Reviews, 241(1), 260–268. 21488902 10.1111/j.1600-065X.2011.01018.x PMC3077798

[jev212457-bib-0054] Saba, E. , Grivel, J. C. , Vanpouille, C. , Brichacek, B. , Fitzgerald, W. , Margolis, L. , & Lisco, A. (2010). HIV‐1 sexual transmission: early events of HIV‐1 infection of human cervico‐vaginal tissue in an optimized ex vivo model. Mucosal Immunology, 3(3), 280–290. 20147895 10.1038/mi.2010.2 PMC3173980

[jev212457-bib-0031] Sahlén, G. E. , Egevad, L. , Ahlander, A. , Norlén, B. J. , Ronquist, G. , & Nilsson, B. O. (2002). Ultrastructure of the secretion of prostasomes from benign and malignant epithelial cells in the prostate. The Prostate, 53(3), 192–199. 12386919 10.1002/pros.10126

[jev212457-bib-0034] Sahlén, G. , Nilsson, O. , Larsson, A. , Carlsson, L. , Norlén, B. J. , & Ronquist, G. (2010). Secretions from seminal vesicles lack characteristic markers for prostasomes. Upsala Journal of Medical Sciences, 115(2), 107–112. 19943818 10.3109/03009730903366067 PMC2853787

[jev212457-bib-0015] Samstein, R. M. , Josefowicz, S. Z. , Arvey, A. , Treuting, P. M. , & Rudensky, A. Y. (2012). Extrathymic generation of regulatory T cells in placental mammals mitigates maternal‐fetal conflict. Cell, 150(1), 29–38. 22770213 10.1016/j.cell.2012.05.031 PMC3422629

[jev212457-bib-0010] Schjenken, J. E. , Glynn, D. J. , Sharkey, D. J. , & Robertson, S. A. (2015). TLR4 signaling is a major mediator of the female tract response to seminal fluid in mice. Biology of Reproduction, 93(3), 68. 26157066 10.1095/biolreprod.114.125740

[jev212457-bib-0058] Selva, K. J. , Kent, S. J. , & Parsons, M. S. (2017). Modulation of innate and adaptive cellular immunity relevant to HIV‐1 vaccine design by seminal plasma. AIDS (London, England), 31(3), 333–342. 27835615 10.1097/QAD.0000000000001319

[jev212457-bib-0018] Sharkey, D. J. , Tremellen, K. P. , Jasper, M. J. , Gemzell‐Danielsson, K. , & Robertson, S. A. (2012). Seminal fluid induces leukocyte recruitment and cytokine and chemokine mRNA expression in the human cervix after coitus. Journal of Immunology, 188(5), 2445–2454. 10.4049/jimmunol.1102736 22271649

[jev212457-bib-0060] Sharkey, D. J. , Macpherson, A. M. , Tremellen, K. P. , & Robertson, S. A. (2007). Seminal plasma differentially regulates inflammatory cytokine gene expression in human cervical and vaginal epithelial cells. Molecular Human Reproduction, 13(7), 491–501. 17483528 10.1093/molehr/gam028

[jev212457-bib-0011] Shima, T. , Inada, K. , Nakashima, A. , Ushijima, A. , Ito, M. , Yoshino, O. , & Saito, S. (2015). Paternal antigen‐specific proliferating regulatory T cells are increased in uterine‐draining lymph nodes just before implantation and in pregnant uterus just after implantation by seminal plasma‐priming in allogeneic mouse pregnancy. Journal of Reproductive Immunology, 108, 72–82. 25817463 10.1016/j.jri.2015.02.005

[jev212457-bib-0041] Simonsen, J. B. (2019). Pitfalls associated with lipophilic fluorophore staining of extracellular vesicles for uptake studies. Journal of Extracellular Vesicles, 8(1), 1582237. 30815239 10.1080/20013078.2019.1582237 PMC6383605

[jev212457-bib-0059] Skibinski, G. , Kelly, R. W. , Harkiss, D. , & James, K. (1992). Immunosuppression by human seminal plasma–extracellular organelles (prostasomes) modulate activity of phagocytic cells. American Journal of Reproductive Immunology (New York, N.Y. : 1989), 28(2), 97–103. 1337434 10.1111/j.1600-0897.1992.tb00767.x

[jev212457-bib-0032] Sullivan, R. , Saez, F. , Girouard, J. , & Frenette, G. (2005). Role of exosomes in sperm maturation during the transit along the male reproductive tract. Blood Cells, Molecules & Diseases, 35(1), 1–10. 10.1016/j.bcmd.2005.03.005 15893944

[jev212457-bib-0024] Tarazona, R. , Delgado, E. , Guarnizo, M. C. , Roncero, R. G. , Morgado, S. , Sánchez‐Correa, B. , Gordillo, J. J. , Dejulián, J. , & Casado, J. G. (2011). Human prostasomes express CD48 and interfere with NK cell function. Immunobiology, 216(1‐2), 41–46. 20382443 10.1016/j.imbio.2010.03.002

[jev212457-bib-0028] Vojtech, L. , Zhang, M. , Davé, V. , Levy, C. , Hughes, S. M. , Wang, R. , Calienes, F. , Prlic, M. , Nance, E. , & Hladik, F. (2019). Extracellular vesicles in human semen modulate antigen‐presenting cell function and decrease downstream antiviral T cell responses. PLoS ONE, 14(10), e0223901. 31622420 10.1371/journal.pone.0223901 PMC6797208

[jev212457-bib-0046] Wang, J. , Ioan‐Facsinay, A. , van der Voort, E. I. , Huizinga, T. W. , & Toes, R. E. (2007). Transient expression of FOXP3 in human activated nonregulatory CD4+ T cells. European Journal of Immunology, 37(1), 129–138. 17154262 10.1002/eji.200636435

[jev212457-bib-0068] Wang, M. , Jia, J. , Cui, Y. , Peng, Y. , & Jiang, Y. (2021). CD73‐positive extracellular vesicles promote glioblastoma immunosuppression by inhibiting T‐cell clonal expansion. Cell Death & Disease, 12(11), 1065. 34753903 10.1038/s41419-021-04359-3 PMC8578373

[jev212457-bib-0050] Willingham, S. B. , Ho, P. Y. , Hotson, A. , Hill, C. , Piccione, E. C. , Hsieh, J. , Liu, L. , Buggy, J. J. , McCaffery, I. , & Miller, R. A. (2018). A2AR antagonism with CPI‐444 induces antitumor responses and augments efficacy to Anti‐PD‐(L)1 and anti‐CTLA‐4 in preclinical models. Cancer Immunology Research, 6(10), 1136–1149. 30131376 10.1158/2326-6066.CIR-18-0056

[jev212457-bib-0003] Wira, C. R. , Fahey, J. V. , Sentman, C. L. , Pioli, P. A. , & Shen, L. (2005). Innate and adaptive immunity in female genital tract: cellular responses and interactions. Immunological Reviews, 206, 306–335. 16048557 10.1111/j.0105-2896.2005.00287.x

[jev212457-bib-0012] Yasuda, I. , Shima, T. , Moriya, T. , Ikebuchi, R. , Kusumoto, Y. , Ushijima, A. , Nakashima, A. , Tomura, M. , & Saito, S. (2020). Dynamic changes in the phenotype of dendritic cells in the uterus and uterine draining lymph nodes after coitus. Frontiers in Immunology, 11, 557720. 33013926 10.3389/fimmu.2020.557720 PMC7516021

[jev212457-bib-0044] Yu, N. , Li, X. , Song, W. , Li, D. , Yu, D. , Zeng, X. , Li, M. , Leng, X. , & Li, X. (2012). CD4(+)CD25 (+)CD127 (low/‐) T cells: a more specific Treg population in human peripheral blood. Inflammation, 35(6), 1773–1780. 22752562 10.1007/s10753-012-9496-8

[jev212457-bib-0067] Zarek, P. E. , Huang, C. T. , Lutz, E. R. , Kowalski, J. , Horton, M. R. , Linden, J. , Drake, C. G. , & Powell, J. D. (2008). A2A receptor signaling promotes peripheral tolerance by inducing T‐cell anergy and the generation of adaptive regulatory T cells. Blood, 111(1), 251–259. 17909080 10.1182/blood-2007-03-081646 PMC2200810

[jev212457-bib-0033] Zhang, X. , Vos, H. R. , Tao, W. , & Stoorvogel, W. (2020a). Proteomic profiling of two distinct populations of extracellular vesicles isolated from human seminal plasma. International Journal of Molecular Sciences, 21(21), 7957. 33114768 10.3390/ijms21217957 PMC7663558

[jev212457-bib-0037] Zhang, X. , Borg, E. G. F. , Liaci, A. M. , Vos, H. R. , & Stoorvogel, W. (2020b). A novel three step protocol to isolate extracellular vesicles from plasma or cell culture medium with both high yield and purity. Journal of Extracellular Vesicles, 9(1), 1791450. 32944179 10.1080/20013078.2020.1791450 PMC7480457

[jev212457-bib-0045] Zheng, Y. , & Rudensky, A. Y. (2007). Foxp3 in control of the regulatory T cell lineage. Nature Immunology, 8(5), 457–462. 17440451 10.1038/ni1455

