# Supplementary material for: Extracellular vesicles from seminal plasma interact with T cells in vitro and drive their differentiation into regulatory T‐cells
Source: J Extracell Vesicles. 2024 Jul 15;13(7):e12457. doi: 10.1002/jev2.12457 (PMC11247398; doi:10.1002/jev2.12457)
Supplement: Supplementary file 1 — Supplementary Information [file JEV2-13-e12457-s001.docx]

**Supplementary Figures Zhang et al**


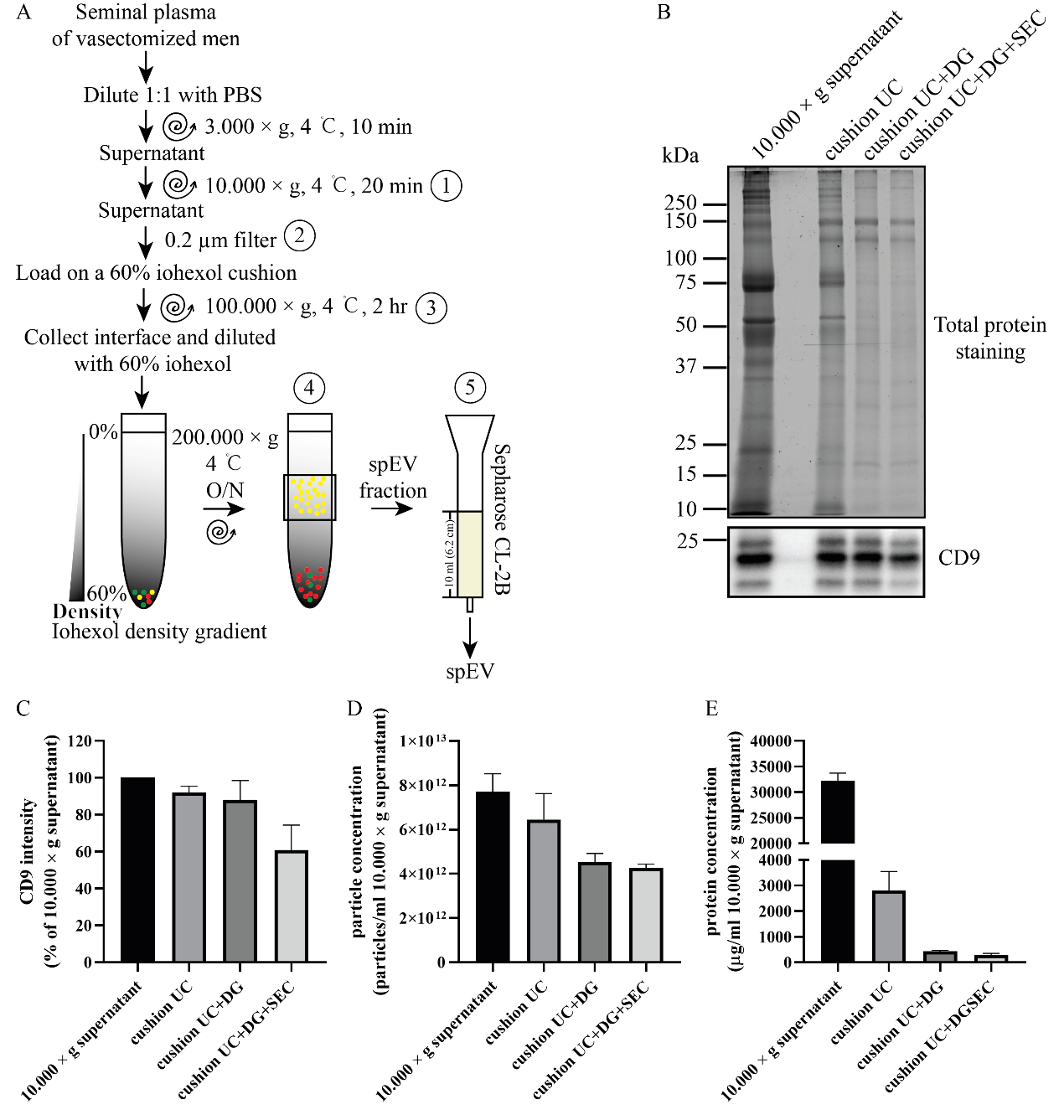


**Supplementary figure 1**. **Isolation of spEVs is efficient and quantitative.** **(A),** Schematic representation of the spEV isolation procedure: 1, removal of any remaining cells and large particles by centrifugation at 10,000×g; 2, filtration to remove any remaining particles > 0.2 µm and potentially contaminating bacteria or fungi; 3, sedimentation by ultracentrifugation at 100,000 × g on top of an iohexol cushion; 4, upward displacement by ultracentrifugation into an iohexol density gradient; 5, removal of iohexol and possibly any other remaining contaminants by SEC. **(B-E),** samples taken after each subsequent isolation step (10,000xg supernatant; cushion UC; combined spEV positive DG fractions; combined spEV positive SEC fractions) were diluted to normalize their volume to that of their originating 10,000×g supernatant. Equal volume samples were then analyzed and compared. (B), analysis by SDS-PAGE followed by total protein staining (B, top panel), or immunoblotting for the presence of CD9 (B, bottom panel). **(C),** The relative amount of CD9 as quantified from immunoblots as shown in B. **(D),** Particle concentration as determined by NTA. **(E),** Total protein as determined by BCA. Values indicate mean ± SD from 3 independent experiments.


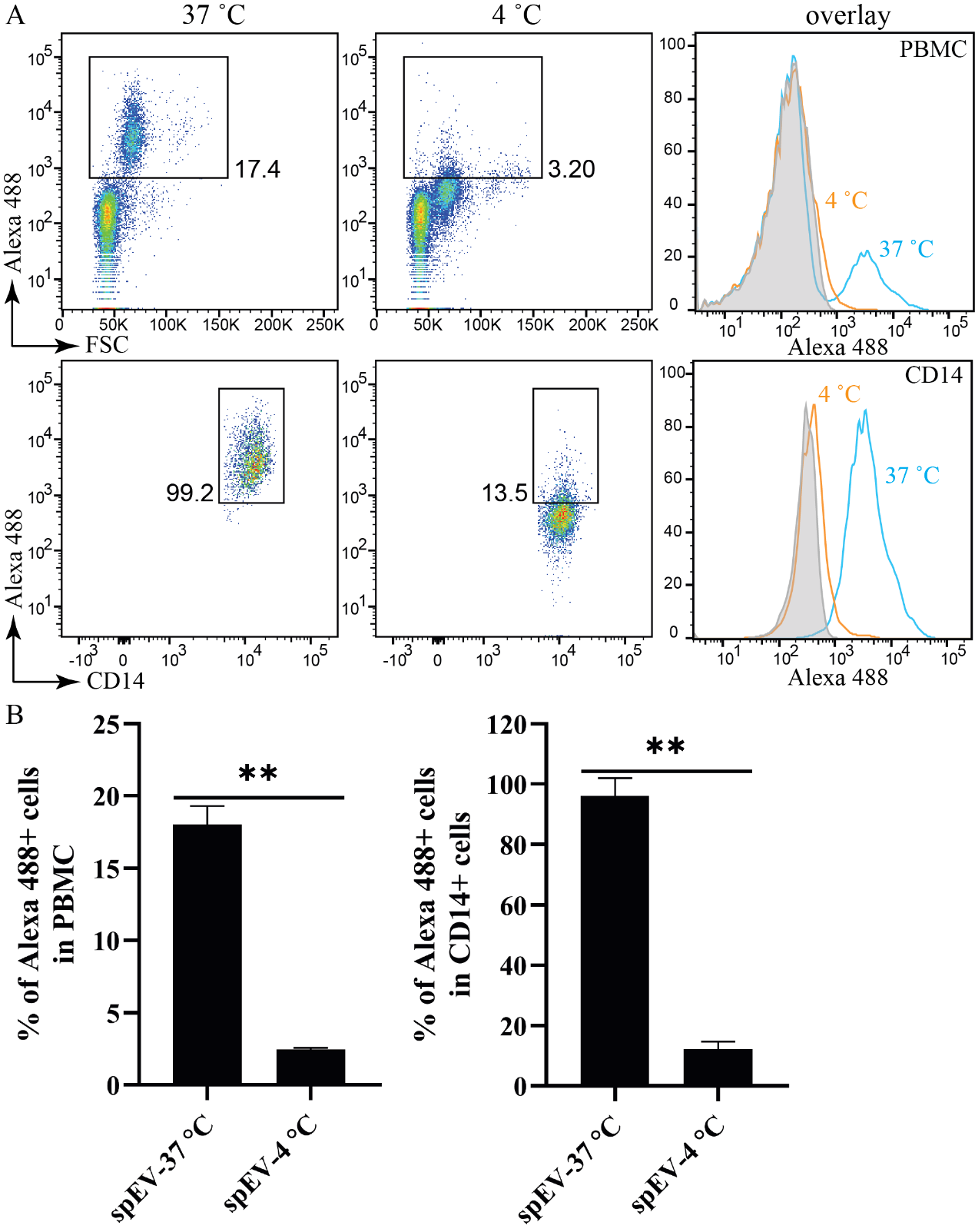


**Supplementary Figure 2. Recruitment of spEVs by PBMCs and CD14+ monocytes is temperature dependent.** **(A),** Representative examples of dot plots and overlayed histograms of Alexa 488-spEV associated total PBMCs and CD14+ monocytes after 6 hr incubation at 37 °C or 4 °C in the presence of Alexa488-spEVs. Background signals in the absence of Alexa 488-spEVs are indicated by the grey areas. **(B),** Quantification of data in A (mean ± SD, n = 3 independent experiments).


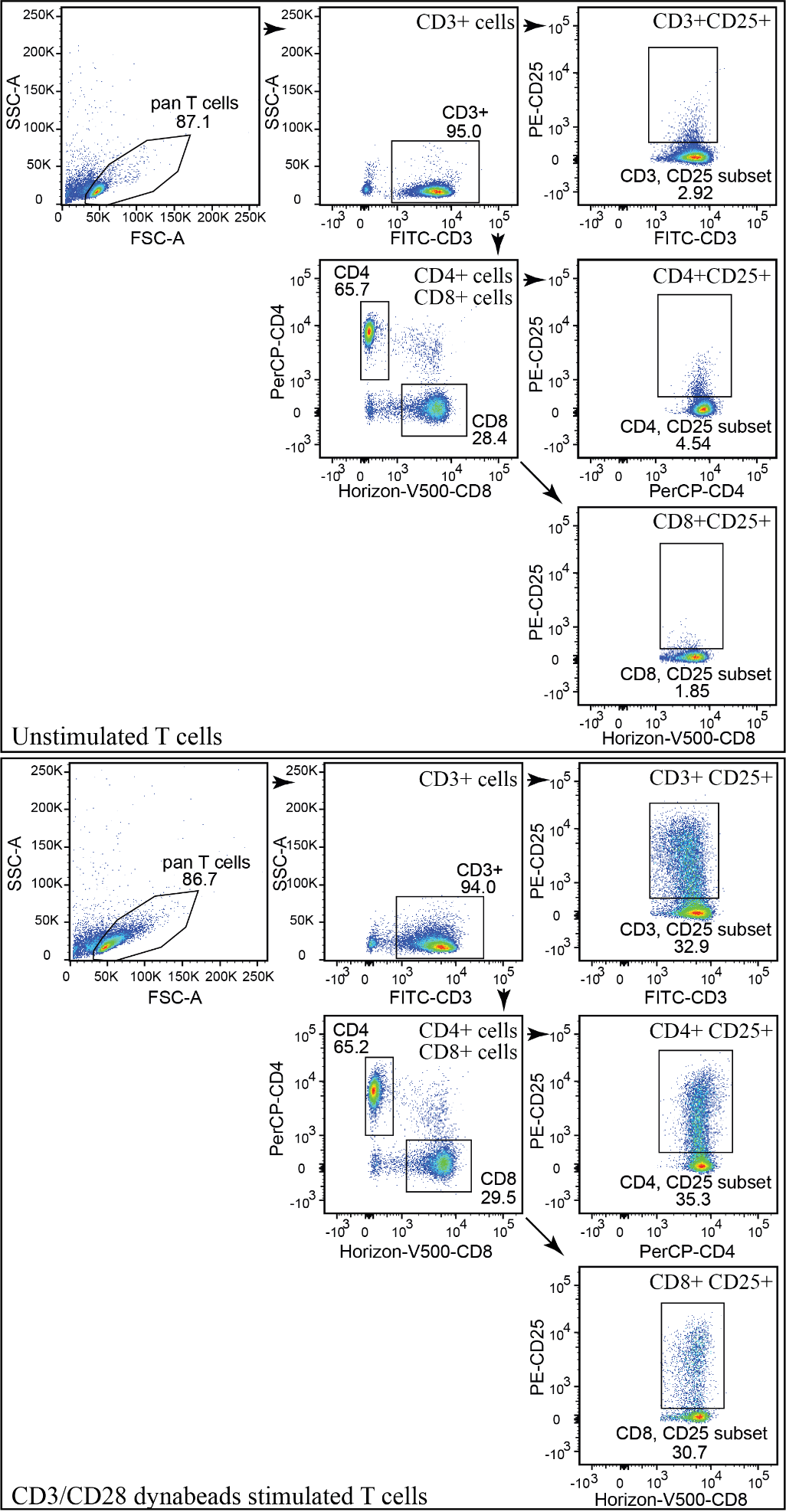
**Supplementary Figure 3. Gating strategy to identify CD25-high cells in CD3+, CD4+, and CD8+ cells.** Isolated pan T-cells were either left unstimulated (upper panel) or stimulated for 16 hr with anti-CD3/CD28 Dynabeads (lower panel). Expression of CD25 on CD3+, CD4+ or CD8+ cells was detected by flow cytometry. Hereto, cells were first gated by side and forward scatter, followed by gating of CD3+ cells. CD4+ and CD8+ cells were gated from CD3+ cells, and finally, CD25-high cells were gated on CD3+, CD4+, and CD8+ cells.


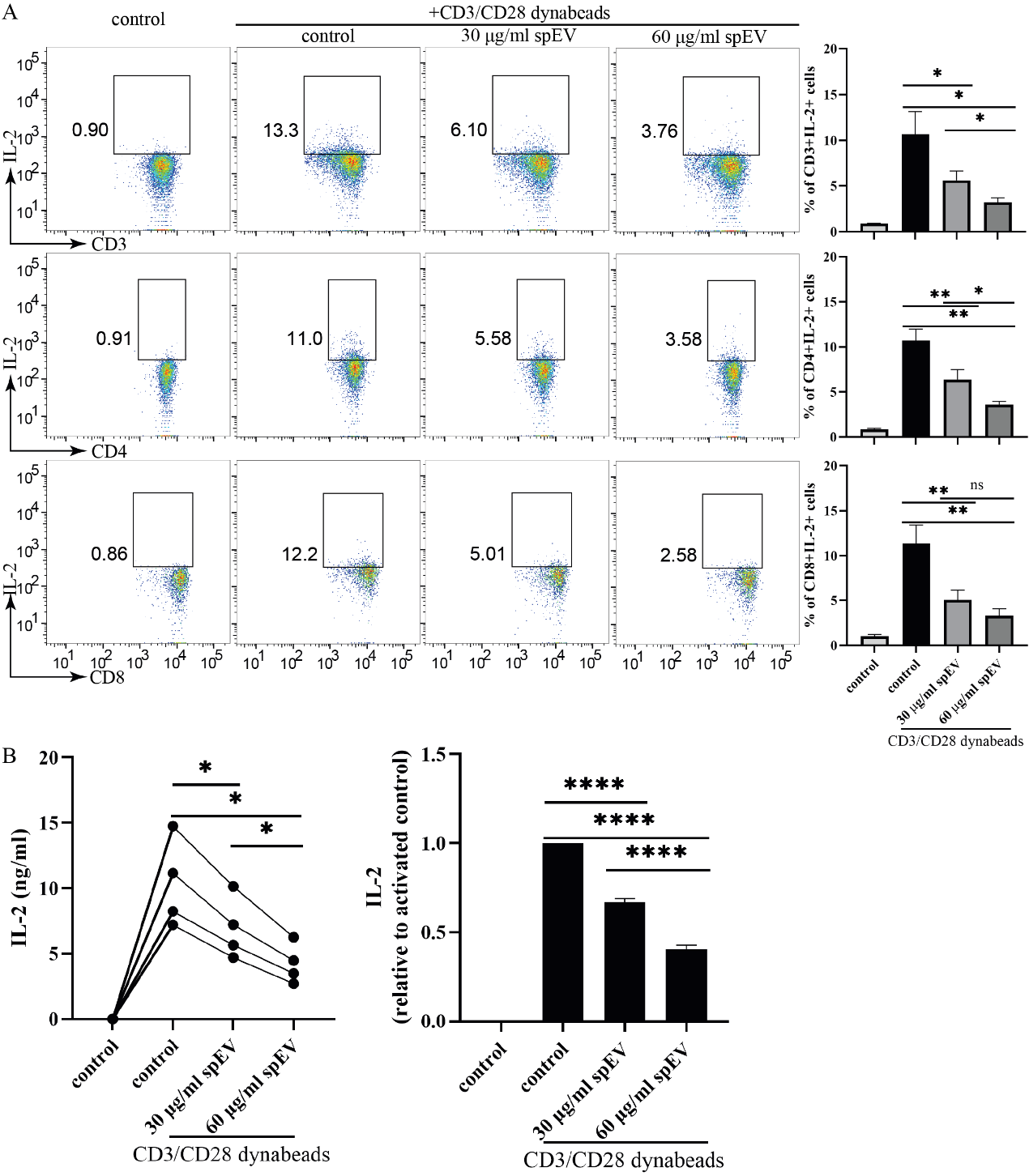


**Supplementary Figure 4. IL-2 production by activated T-cells is inhibited by spEVs in a concentration dependent manner**. Isolated pan T-cells were left unstimulated or stimulated for 16 hr with anti-CD3/CD28 Dynabeads, either in the absence (control) or presence of 30 µg/ml or 60 µg/ml spEVs. **(A),** IL-2 in CD3+, CD4+ and CD8+ cells was detected by flow cytometry (mean ± SD, n = 4 independent experiments). **(B),** IL-2 in the cell culture medium was determined by ELISA and is depicted as ng/ml (left panel) and percentage relative to control (mean ± SD, n = 4 independent experiments) (right panel).


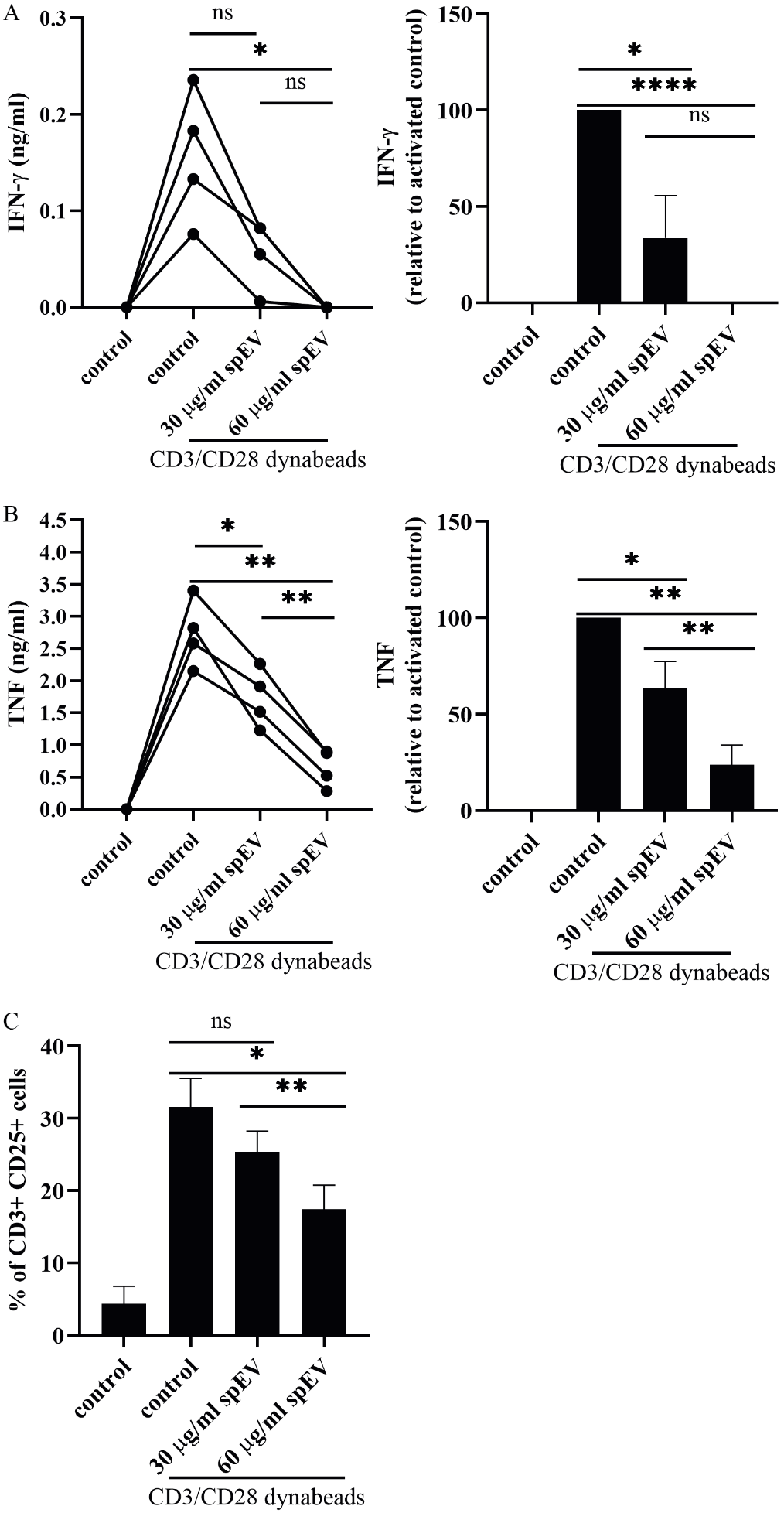


**Supplementary Figure 5. IFN-γ and TNF production and CD25 expression by activated T-cells is inhibited by spEVs in a concentration dependent manner**. Isolated pan T-cells were stimulated for 16 hr with anti-CD3/CD28 Dynabeads, either in the absence (control) or presence of 30 µg/ml or 60 µg/ml spEVs. (**A and B**), IFN-γ **(A)** and TNF **(B)** in the cell culture medium were determined by ELISA. (C), CD25 expression on CD3+ cells was analyzed by flow cytometry (mean ± SD, n = 4 independent experiments). Note that IFN-γ was undetectable after T-cell stimulation in the presence of 60 µg/ml spEVs, while only minor amounts were still detectable in comparable experiments shown in Figure 6 and supplementary Figure 6. This discrepancy is probably due to slight differences among spEV isolates and/or T-cell donors.


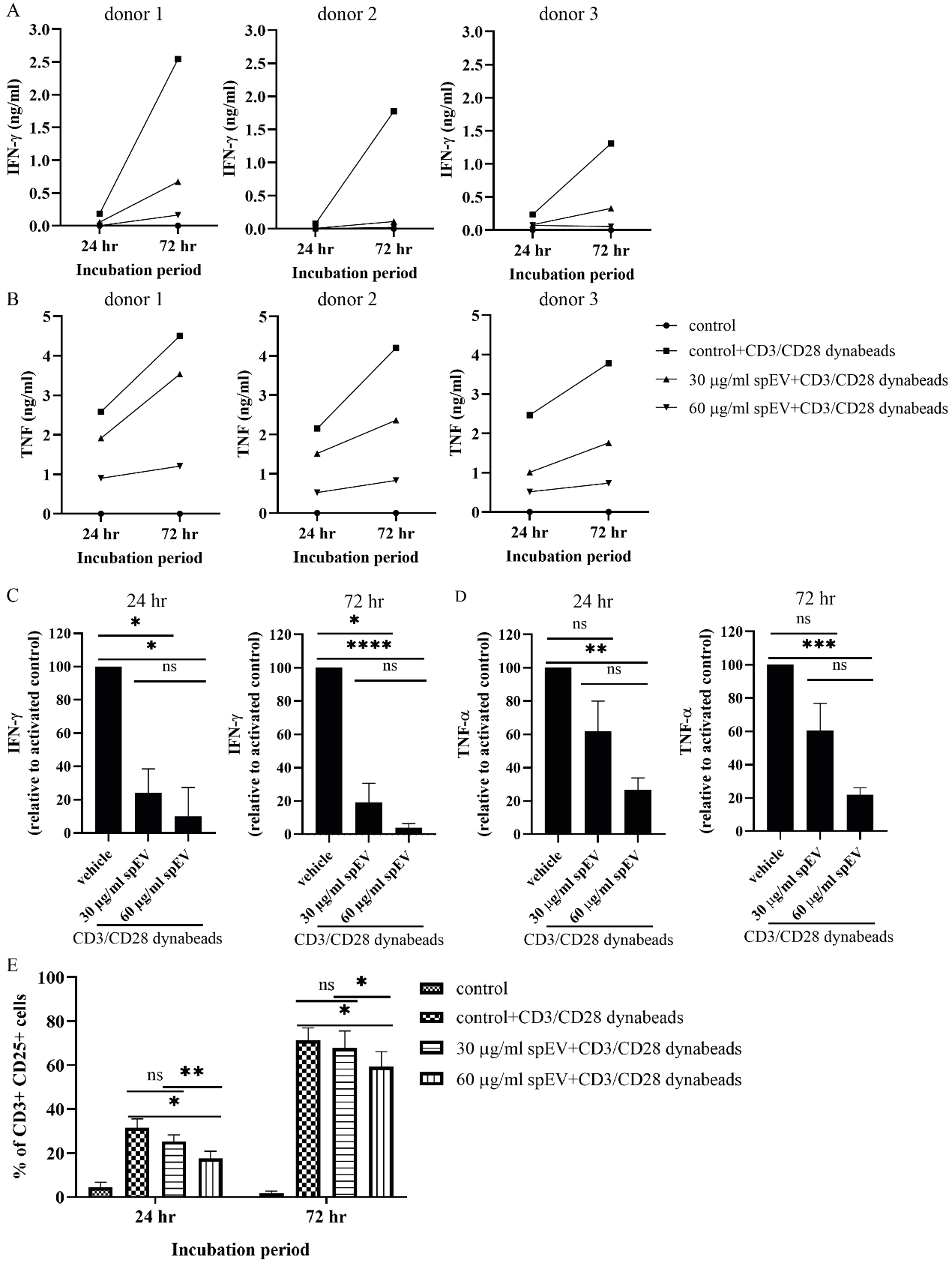


**Supplementary Figure 6. Sustained interference by spEVs on T-cell cytokine production and CD25 expression.** Isolated pan T-cells were stimulated for 24 hr or 72 hr with anti-CD3/CD28 Dynabeads in the absence (control) or presence of 30 µg/ml or 60 µg/ml spEVs. **(A-D),** IFN-γ and TNF in the cell culture medium were analyzed by ELISA. The absolute amounts of IFN-γ and TNF differed between T-cell donors (**A and B**), but the relative inhibition of cytokine secretion by spEVs was reproducible and consistent (**C and D**). **(E),** Percentage of gated CD25-high activated CD3+ T-cells, as defined by flow cytometry (mean ± SD, n = 3 independent experiments).


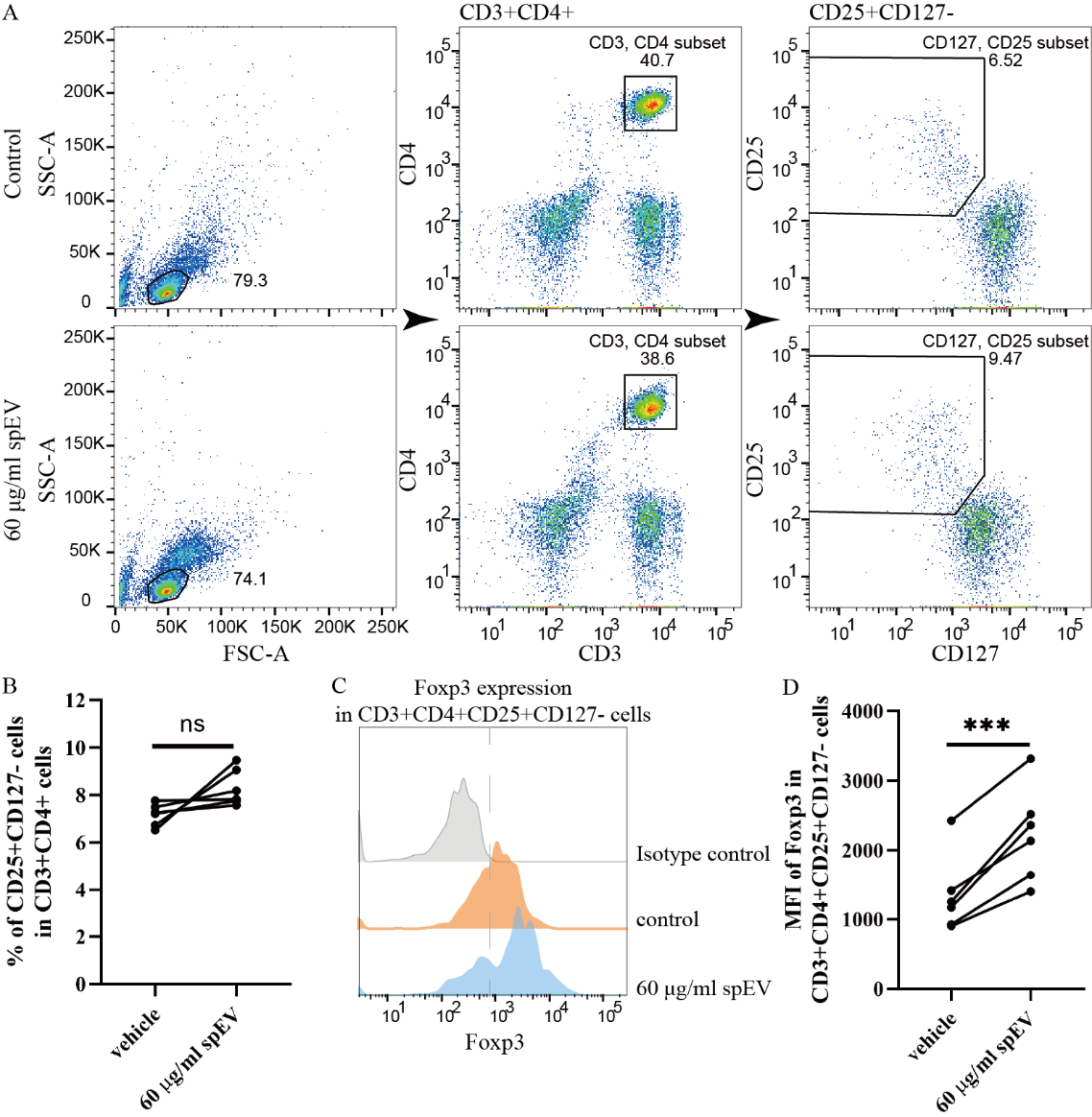


**Supplementary Figure 7. spEVs drive development of Treg in PBMCs.** PBMCs were cultured for 3 days in the absence or presence of 60 µg/ml spEVs and then analyzed by flow cytometry. **(A),** Gating of CD3+CD4+CD25+CD127-) T-cells. **(B),** Gated CD25+CD127- T-cells as in **(A)** are presented as percentage of total CD3+CD4+ T-cells. **(C),** Representative histograms indicating Foxp3 expression in CD3+CD4+CD25+CD127- gated cells. **(D),** Quantification of data as in **(C)** as MFI of Foxp3+ for 6 independent experiments.


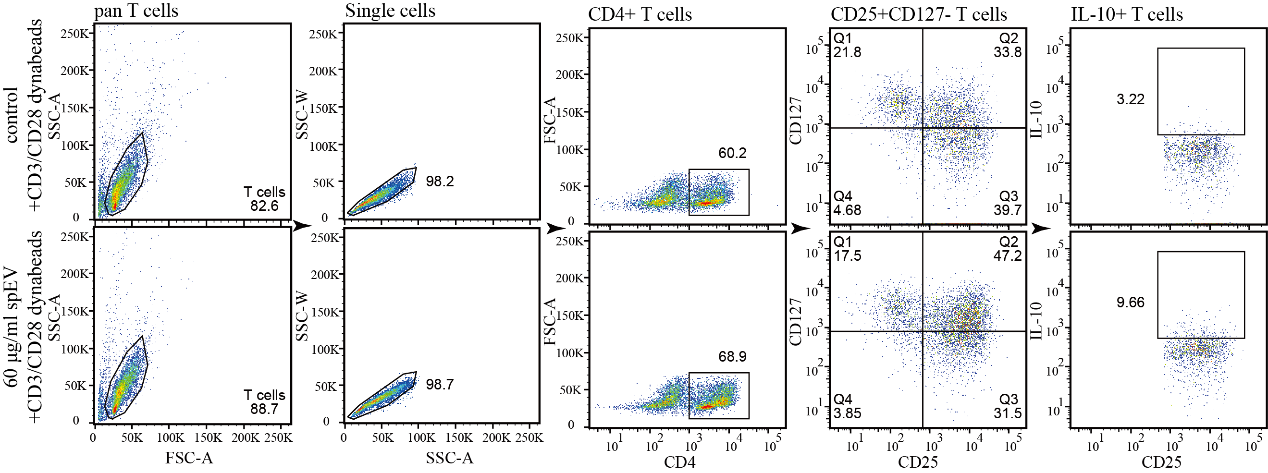


**Supplementary Figure 8. Gating strategy to measure IL-10 in Tregs.** Isolated pan T-cells were activated with anti-CD3/CD28 Dynabeads either in the absence or presence of spEVs, fixed, permeabilized, immunolabelled for IL10, and analyzed by flow cytometry. Hereto, cells were first gated on forward and side scatter, followed by the selection of single cells (SSC-A vs SSC-H). CD4 T-cells were selected based on the cell surface expression of CD4. Tregs were selected based on the cell surface expression of CD25 and the lack of CD127 expression (Q3). CD4+CD25+CD127-IL10+ cells were gated as shown.


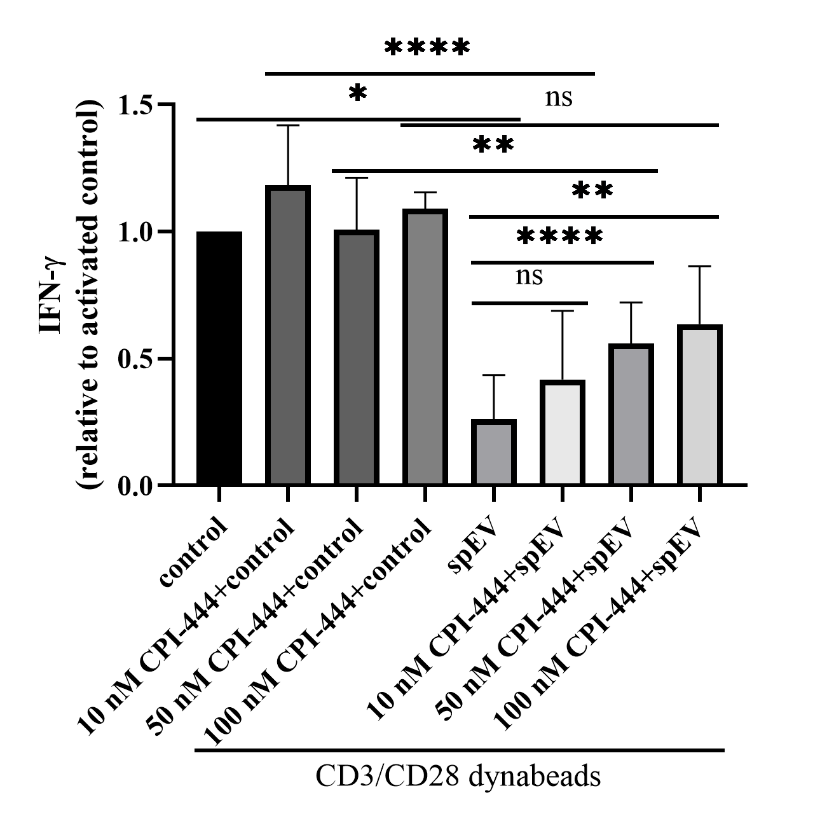


**Supplementary Figure 9. CPI-444 reverses the inhibitory effects of spEVs on T-cell IFN-γ production in a concentration dependent manner.** Isolated pan T-cells were stimulated for 16 hr with anti-CD3/CD28 Dynabeads in the absence (control) or presence of 60 µg/ml spEVs and 0, 10, 50, or 100 nM CPI-444, as indicated. IFN-γ in the cell culture medium was then analyzed by ELISA and plotted as % of the control condition (mean ± SD, n = 3 independent experiments).
